# Neuropsychiatric Outcomes in Children and Adolescents With Perinatally Acquired HIV: A Systematic Review and Meta-Analysis

**DOI:** 10.1097/QAI.0000000000003595

**Published:** 2025-03-10

**Authors:** Rebecca H. Horton, Amy Mcintosh, Edoardo G. Ostinelli, Elinor Harriss, Mina Fazel

**Affiliations:** a Department of Paediatrics, University of Oxford, Oxford, United Kingdom;; b Norwich Medical School, University of East Anglia, Norwich, United Kingdom;; c Department of Psychiatry, University of Oxford, Oxford, United Kingdom;; d Oxford Precision Psychiatry Lab, NIHR Oxford Health Biomedical Research Centre, Oxford, United Kingdom;; e Oxford Health NHS Foundation Trust, Warneford Hospital, Oxford, United Kingdom;; f Bodleian Health Care Libraries, University of Oxford, Oxford, United Kingdom; and; g Children's Psychological Medicine, Oxford University Hospitals NHS Foundation Trust, Oxford, United Kingdom.

**Keywords:** HIV, perinatally acquired HIV, pHIV, mother-to-child transmission, child development

## Abstract

**Objective:**

The objective of this study is to define the neuropsychiatric challenges including developmental delay, cognitive impairment, and psychiatric illness faced by children with perinatally acquired HIV.

**Data sources:**

Nine databases were searched on May 30, 2023: MEDLINE, Embase, and PsycINFO (all through Ovid SP); CINAHL and Child Development and Adolescent Studies (through EBSCO); the Web of Science Core Collection; Scopus; ProQuest Dissertations and Theses Global; and WHO Global Index Medicus. No limits were applied. Search strategies incorporated keywords and thesaurus headings to describe children and adolescents aged 0–25 years with perinatally acquired HIV and terms to describe the spectrum of neuropsychiatric impairment.

**Study selection:**

Entries were reviewed by 2 independent reviewers. Studies were included if they involved a population of children with perinatally acquired HIV and investigated a neurologic or psychiatric outcome.

**Main outcome measures:**

Hypothesis that children with pHIV would have more neuropsychiatric challenges than children without pHIV was formulated before the study. Main outcome measures include incidence and severity of cognitive impairment, developmental delay, and psychiatric illness in children with pHIV.

**Results:**

Forty-five studies on cognitive impairment were included of which 8 studies were included for meta-analysis and demonstrated a standardized mean difference of −0.508 where children without HIV had higher cognitive scores (95% CI: −0.7903 to −0.2272). In total, 15 studies on developmental delay were included, of which 9 were included for meta-analysis and demonstrated, for motor delay, a standardized mean difference (SMD) of −0.794 where children without HIV achieved higher motor function scores (95% CI: −0.9986 to −0.590) and for cognitive delay an SMD of −0.697 where children without HIV achieved higher cognitive function scores (95% CI: −0.976 to −0.417). In total, 39 studies on psychiatric illness were included with an odds ratio for anxiety and depression of 1.105, suggesting that children with HIV had slightly higher odds of developing anxiety or depression, however, this result was not significant (95% CI: 0.778 to 1.571).

**Conclusions:**

Children with perinatally acquired HIV may have a greater cognitive impairment, motor and cognitive delay, and would likely benefit from tailored approaches to improve their outcomes.

## Abstract 2

Supplemental Digital Content is Available in the Text.

## INTRODUCTION

The course of childhood HIV has changed dramatically in the past few decades, primarily because of the availability of combination antiretroviral therapy (cART). More children with perinatally acquired HIV are reaching adolescence and with this comes the emergence of a chronic disease syndrome including pulmonary, musculoskeletal, renal, and cognitive symptoms.^1^ This seems distinct to the disease seen in adults, because symptoms affect growth and development during childhood. This alters the course of neurologic and musculoskeletal sequelae in particular. Coupled with this are the psychological and social effects of living with HIV. As this cohort grows up, it, therefore, becomes essential to better understand and characterize what support these children and young people need.

Despite impressive increases in pregnant women receiving ART (from 44% in 2010 to 82% in 2018), there still exists a large cohort of children who acquire HIV from vertical transmission in utero from their mothers.^2^ In the absence of ART, 15%–45% of HIV-positive women transmit the infection to their babies in utero, intrapartum, or during breast feeding.^3^ The burden of pediatric HIV, therefore, remains high, with an estimated 2.5 million children worldwide living with the virus, 90% of whom live in sub-Saharan Africa.^4^ Of these children, only approximately 57% of affected children receive ART, and when they do, it is often commenced late in the disease course.^4^

Neurodevelopmental impairments are an important but largely unaddressed problem for children in low-income countries,^5^ exacerbated by their prior absence from the sustainable development goals (SDGs), only added recently for the 2030 SDGs. Because HIV is a neurotropic and a lymphotropic virus, and central nervous system (CNS) invasion occurs early in the disease course, the window of opportunity in which to minimize the reservoir of virus in the CNS is likely short.^6–8^ Early pre-ART disease progression may be associated with irreversible neuronal damage.^9^ The burden of neuropsychiatric disease in this population is likely a combination of social and biologic factors. This may also manifest as mental illness, which also often remains largely unrecognized regardless of being a leading cause of disability.

Despite marked and valuable progress, children with pHIV remain a population with high unmet need. There is likely a large burden of neurologic and psychiatric illness in this population that when unrecognized and untreated leads to avoidable disability. Multiple factors are at play across the whole biopsychosocial realm of illness. Critically appraising the existing evidence is crucial to better map and understand the needs of children with HIV to support them to reach their full potential.

## METHODS

The study protocol was registered on PROSPERO (CRD42020159159).

### Search Strategy

A full search strategy is available in the Supplemental Digital Content (see http://links.lww.com/QAI/C418).

### Eligibility Criteria

Selection criteria are outlined in Table 1. Studies with HIV-affected controls (perinatally HIV-exposed uninfected (PHEU) or perinatally HIV-unexposed, uninfected (PHU) living in HIV-affected households), healthy PHU controls, or population norms were included. Where studies included children <10 years, perinatal transmission was presumed. Studies were eligible for meta-analysis if they contained a control group of either HIV-exposed uninfected children or HIV-unaffected children and had available raw data (for instance, absolute scores of development rather than relative scores) available for use in the meta-analysis. Where data were not available, study authors were contacted to obtain this information. No further information was obtained.

**TABLE 1.** Inclusion and Exclusion Criteria

Criteria	Inclusion	Exclusion
Population	1. Perinatally HIV-1 infected 2. Child, adolescent aged 0–25 yrs	1. Behaviorally acquired HIV 2. HIV-exposed, uninfected (children whose mothers had HIV during pregnancy or lactation but did not contract HIV) 3. Transfusion-acquired HIV
Outcomes	Primary outcome 1. Any neurologic or psychiatric outcome	1. Primary outcome is not a neurologic or psychiatric outcome
Study design	1. Observational (cross-sectional or longitudinal) with or without controls 2. Cohort, case–control 3. Case series with >5 participants	1. Case reports with <5 participants
Source	1. Primary sources	1. Excluding conference abstracts, theses 2. Secondary sources (textbooks)

### Outcomes

Three primary outcomes were identified:

1. Difference in developmental achievement score measured using standardized assessment tools between HIV+ and HIVEU children (continuous).
2. Difference in cognitive function measurements between HIV+ and HIV-uninfected children (continuous).
3. Difference in incidence in diagnosis of anxiety and/or depression between children and young people with perinatally acquired HIV and exposed-uninfected or unexposed-uninfected controls (binary).

Secondary outcomes were the type and nature of neuropsychiatric outcome measured.

### Study Appraisal

Study Appraisals—Newcastle Ottawa scoring system was used, see Supplemental Digital Content (see http://links.lww.com/QAI/C418). All studies were included because the aim of this review was to gain a broad understanding of children living with HIV because limiting inclusion to larger, newer studies may have narrowed our view of the experiences of these children and how they have changed over time.

### Synthesis Without Meta-Analysis

We considered and appraised quantitative data regarding the 3 primary outcome measures: cognitive impairment, developmental delay, and psychiatric illness.

### Meta-Analysis

We performed a series of random effects (inverse variance) meta-analyses on our 3 coprimary outcomes. For continuous outcomes, where different rating scales were used, we estimated the pooled effect size using a standardized mean difference (SMD). For binary outcomes, we summarized the pooled effect size using an odds ratio (OR). For all the pairwise meta-analyses, we used DerSimonian–Laird estimator for tau^2^, the Jackson method to estimate the confidence interval of tau^2^ and tau. We estimated the 95% prediction interval based on t-distribution to account for the impact of heterogeneity on the summary estimates.

### Patient and Public Involvement

We held a focus group of 12 young people with perinatally acquired HIV who were part of the Chiedza trial in Harare, Zimbabwe.^10^ They were aged between 11 and 18 years and 3 guardians also attended and contributed to the discussion. The group was chaired by Dr Nyasha Dzavakwa in person with Drs Rebecca Horton and Mina Fazel remotely through MS Teams videoconferencing. Translation between English and Shona (the first language of the attendees) was done contemporaneously by Dr Nyasha Dzavakwa. The questions put to the participants are given in the Supplemental Digital Content (see http://links.lww.com/QAI/C418).

## RESULTS

### General Study Characteristics

In total, 2278 potentially eligible study titles were found by the search after deduplication, and the titles of these were screened for inclusion. In total, 912 abstracts were subsequently screened for inclusion. Out of 337 potentially eligible abstracts, we included 99 studies meeting the inclusion criteria comprising 16,080 children with pHIV. The PRISMA flow diagram is available in the Supplemental Digital Content (see http://links.lww.com/QAI/C418). Of these studies, 28 (8%, 6155 participants) contributed with quantitative data to 1 or more meta-analyses. Eligible studies consisted of both cross-sectional and cohort studies. Both HIV-exposed, uninfected children, who had in utero exposure to HIV but never seroconverted, and HIV-unexposed children, were included as controls. It is notable that, throughout all the studies, those conducted in the post-ART era included populations with significantly higher rates of ART than what has been reported globally by UNICEF.^4^

### Developmental Delay

#### Study Characteristics

**FIGURE 1.**
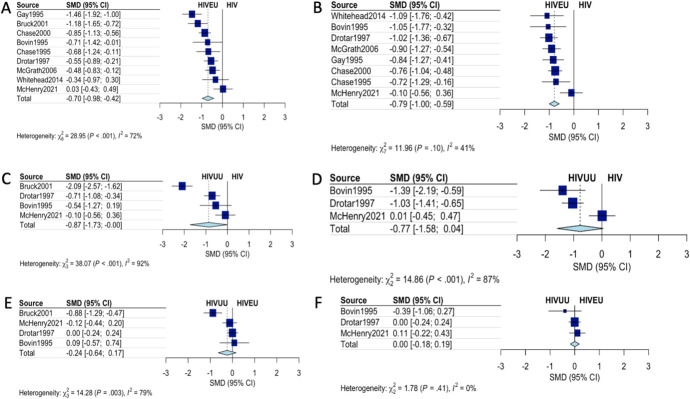
Development delay in children infected with HIV. Standardized mean difference (SMD) more than 0 favors the group on the right-hand side of the groups in terms of neurodevelopment. A, Cognitive development of HIV+ compared with children with HIVEU. B, Motor development HIV vs HIVEU. C, Cognitive development HIV vs HIVUU. D, Motor development HIV vs HIVUU. E, Cognitive development HIVEU vs HIVUU. F, Motor development HIVUU vs HIVEU-exposed, uninfected HIVUU—Unexposed uninfected.

Fifteen studies investigated developmental delay, of which 9 (60%) contributed data to the meta-analysis comprising 356 children infected with HIV, 1018 children exposed to HIV but not infected, and 273 children who were unexposed and uninfected (Fig. 1). Studies were conducted in Brazil (1), Canada (1), Kenya (2), South Africa (3), Tanzania (2), United States (2), Zaire (1), and Zimbabwe (1). Twelve (80%) of the 15 studies were conducted in low- and middle-income countries. Data summarizing the included studies are presented in Table 2.

**TABLE 2.** Characteristics and Findings of Studies Investigating Developmental Delay

	Study	Study Design	Location	Number of Participants			Key Findings	ART Regime
				HIV+	HIVEU	HIVUU		
Not on ART	Minden 1992	Cohort	Canada	9	9	0	HIV+ children performed significantly worse. More marked as age progressed. Six died from AIDS	N/a
	**Chase 1995**	**Cohort**	**United States**	**24**	**27**	**0**	**50% HIV+ children moderate–severe neurodisability. 18% HIVEU children. Infected group declining mental skills over time**	N/a
	**Gay 1995**	**Cohort**	**Haiti**	**28**	**98**	**0**	**Cognitive and motor delay increasing for 2 years of life**	N/a
	**Boivin 1995**	**Cohort**	**Zaire**	**14**	**20**	**16**	**HIV-positive children scored significantly lower than HIV-negative children and HIVUU**	N/a
	**Drotar 1997**	**Cohort**	**Uganda**	**79**	**241**	**116**	**HIV+ group 44% motor delay, 35% cognitive delay vs 10% each category both other groups**	N/a
	**Chase 2000**	**Cohort**	**United States**	**77**	**344**	**0**	**Prevalence of delay increased for 2 yrs**	N/a
	**Bruck 2001**	**Cohort**	**Brazil**	**43**	**40**	**67**	**Highly significant differences noted among HIV-infected children**	N/a
	**McGrath 2006**	**Cohort**	**Tanzania**	**40**	**145**	**0**	**Testing positive in first 21 days of life is associated with a 14.9 times higher rate of being delayed in terms of cognitive function**	N/a
	Kandawasvika 2011	Cohort	Zimbabwe	65	118	287	Rate of neurodevelopmental impairment double HIV+ than uninfected that increased over time	N/a
On ART	Ferguson G 2009	Cohort	South Africa	51	0	35	66% HIV+ significant motor delay HIV+, 5.7% significant motor delay HIVUU	67% on ART after disease progression
	Brahmbhatt 2014	Cohort	Uganda	116	105	108	Longer duration ART, less DD. HIV+ children significantly more delay in all areas	ART use as per WHO criteria −44% of children
	**Whitehead 2014**	**Cohort**	**South Africa**	**27**	**29**	**0**	**Delay in HIV+ gone by age 2 yrs**	**All children on ART.**
	Benki-Nugent 2017	Cohort	Kenya	73	0	92	HIV-infected infants on ART had delays in developmental milestone attainment compared with HUU. Poor 6-month post-ART responses predicted greater delay	All children on ART
	Odejayi R 2019	Cross-sectional	South Africa	71	0	0	Visual perception was the most affected in this sample with an average delay of between 11 and 17 months found	All children on ART
	**McHenry 2021**	**Cohort**	**Kenya**	**24**	**74**	**74**	**The only neurodevelopmental difference found among groups was that children who were HIV+ had higher receptive language scores**	**All children on ART**

Bold print signifies a study included in meta-analysis.

#### Meta Analysis

Nine studies were included in the meta-analysis. Children perinatally infected with HIV were delayed in motor (SMD of −0.794 – (95% CI: −0.9986 to −0.590); prediction interval −1.3120; to −0.2764) and cognitive (SMD of −0.697 (CI: −0.976 to –0.417); prediction intervals −1.6004 to 0.2063) skill acquisition as measured by scoring systems compared with children who were perinatally exposed but uninfected by HIV. They were also more likely to be classified as developmentally delayed both in cognitive (OR 3.687 95% CI: 2.55 to 5.31 *P* < 0.001, prediction interval 2.373 to 5.729) and motor (OR 4.07, 95% CI: 2.439 to 6.814, *P* < 0.001, prediction interval 1.222 to 13.595) outcomes compared with their exposed but uninfected counterparts.

**FIGURE 2.**
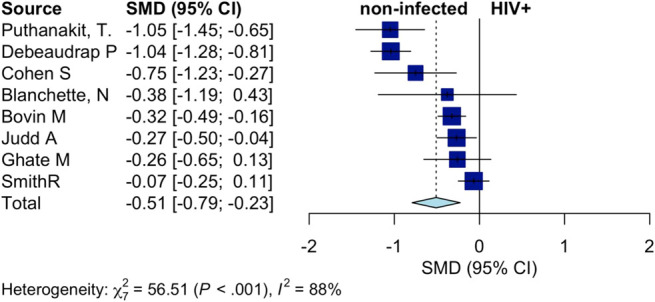
Cognitive performance in HIV-infected vs uninfected children. Overall, children infected by HIV performed more poorly on measures of cognitive performance than those not infected. There are high levels of heterogeneity between studies. Only school-aged children were included in the meta-analysis. Standardized mean difference more than 0 favors the group on the right.

The comparison between HIV-unexposed children and HIV-exposed uninfected children did not exclude null effects in both motor (SMD 0.0045, 95% CI: −0.1791 to 0.1881]) and cognitive domains (SMD −0.2378, 95% CI: −0.6416 to 0.1661). There was large variation in effect size between studies (see Fig. 2) that may reflect different study thresholds for prescribing ART.

#### Synthesis Without Meta-Analysis

The studies conducted in the pre-ART era (7 studies, 46%) all demonstrated significant motor and cognitive delay in HIV-infected children. Motor delay presents early and persists, whereas cognitive delay tends to develop later and increases throughout the first 2 years of life.^11–14^ HIV-infected groups had a high attrition rate because of the death of study participants.^14^ Earlier studies (pre-2009), and studies conducted in low- and middle-income countries report higher rates of more significant developmental delay and were less likely to report use of ART at diagnosis.

Eight studies were conducted in the post-ART era. In 2 of these, ART was only given to children with disease progression and these children had significantly delayed development compared with their uninfected peers.^15,16^ Giving ART early in the disease course reduces the extent of developmental delay, and a poor response to ART at 6 months correlates with poorer developmental outcomes.^17,18^ Whitehead et al (2014) describe a cohort of children on highly active antiretroviral therapy (HAART) who were not significantly delayed in development compared with their peers at 2 years of age.^19^

The timing of HIV infection seems to affect development. Babies who tested HIV positive on day 1 of life had poorer developmental outcomes than babies who tested HIV negative on day 1 of life but positive at day 21 of life^20^ because of likely transmission through breast milk.

### Cognitive Impairment

#### Study Characteristics

In total, 45 studies were eligible for inclusion, of which 8 (17%) contributed data to the meta-analysis, including 1149 HIV-infected children and 1341 noninfected controls. Control groups consisted of children who were either exposed to HIV in utero, or who lived with a family member who had HIV and were combined in a single group for meta-analysis. Studies were conducted in Brazil (1), Canada (1), Cameroon (1), England (4), France (1), India (1), Kenya (1), Malawi (1), Myanmar (1), Poland (1), South Africa (1), The Netherlands (6), Uganda (2), United States (16), Zambia (3), and Zimbabwe (1).

Study characteristics are described in Table 3.

**TABLE 3.** Characteristics and Findings of Studies Investigating Cognitive Impairment

Study	Study Design	Location	ART Regime	Age in years (mean)	Number of Participants			Key Findings
					HIV+	HIVEU	HIVUU	
Papola 1994	Cross-sectional	United States	78 out of 90 children (83/90 completed all assessments)	5 to 14	83	0	0	63% in special education. Emotional and behavioral disorders affected 42% of children, 50% developmental language impairment. More than 50% lower expected intelligence
Tardieu 1995	Cross-sectional	United States	88% on zidovudine for 4 yrs	>6 (9.5)	33	0	0	21 out of 33 children performing normally at school. School failure rate higher than expected. Longer ART use associated with better performance
Fishkin 2000	Cross-sectional	United States	None	3 to 5	40	0	40	Gross cognitive deficits are not evident among preschool children infected with HIV relative to matched controls. Evidence of focal deficits
**Blanchette 2002**	**Cross**-s**ectional**	**Canada**	**92% on combination regime**	**6 to 15**	**13**	**0**	**11**	**No significant differences between groups, but poorer academic attainment in both groups compared with population averages**
Kullgren 2004	**Cross-sectional**	**United States**	**92% on ART**	3 to 16 (6.69)	67	0	0	Mean cognitive performance 1 SD lower than the mean
Smith 2006	Cohort	United States & Puerto Rico	Unknown	3 to 7	117	422	0	HIV+ group and class C status scored significantly lower in all domains of cognitive development, than those without an AIDS-defining illness and HIVEU group
Koekkoek 2008	Cohort	Netherlands	18 on HAART	6 to 17 (9.46)	22	0	0	Global IQ similar between groups but working memory and executive function most sensitive to HIV infection. Higher CD4 at initiation of HAART and longer duration of treatment were associated with better working memory
Brackis-Cott 2009	Cohort	United States	Unknown	9 to 16	43	0	0	29% less than 10th percentile on vocabulary testing. 24% less than 10th percentile on cognitive functioning. Many youth held back school years or placed in special education
Paramesparan 2010	Cross-sectional	England	All children on ART	16 to 25	6	0	0	High rates of asymptomatic cognitive impairment in HIV+ group (67%) compared with older subjects (19%)
**Puthanakit 2010**	**Cross-sectional**	**Thailand**	**34 on ART**	**9.3**	**39**	**40**	**42**	**HIV+ group had a significantly higher risk of a poor cognitive outcome, 79% of HIV+ children had a less than normal IQ & 20% were in less than age-appropriate school grade, compared with 2% of HIVEU group and no children in the HIVUU group**
Rice 2012	Cohort	United States	72% on HAART	7 to 16	306	162	0	35% all children >1SD below average on language score. Overall rates of language impairment comparable between groups
**Smith 2012**	**Cohort**	**United States**	**Unknown**	**7 to 16**	**558**	**200**	**0**	**HIV disease severity consistently associated with poor cognitive and/or adaptive outcomes**
Abubakar 2013	Cohort	Kenya	None	6–35 mo	31	17	0	HIV+ children had lower psychomotor functioning at baseline than HIVEU children
Llorente 2014	Cohort	United States	All children on ART	8 to 12	76	85	0	Significant differences in several measures of executive function between HIV+ and HIVEU.
Garvie 2014	Cohort	United States	All children on ART, 80% of whom were HAART	7–16 yrs	295	167	0	Cognitive and achievement scores lower than population means, no differences in cognition between groups. HIV+ children scored lower on achievement
Kandawasvika 2015	Cohort	Zimbabwe	11 children on cART	7 to 14 (median 7.9)	32	121	153	There was no difference in the prevalence of cognitive impairment by HIV status
Ashby 2015	Cross-sectional	England	79% on cART	16 to 25	33	0	14	No differences between cognitive scores or IHDS scores between groups. Worse memory score in the HIV-infected group. Basal ganglia inflammatory metabolites significantly higher in the HIV+ group
**Cohen 2015**	**Cross-sectional**	**Netherlands**	**All children on cART**	**8 to 18**	**35**	**0**	**37**	**HIV+ group had lower IQ scores than controls. Controls also had lower IQ scores than Dutch average**
**Ghate 2015**	**Cross-sectional**	**India**	**Unknown**	**6 to 12**	**50**	**0**	**50**	**No differences between groups**
Linn 2015	Cohort	Myanmar	All children on ART	10.7	28	0	31	HIV-infected children performed poorly across all tests, with significant group differences in executive function, visuospatial reasoning, fine motor dexterity, and visual motor integration
Zielinska 2015	Cross-sectional	Poland	All children on ART	6 to 18	50	0	0	HIV+ demonstrated deficits in EF.
Ezeamama 2016	Cohort	Uganda	45 children on ART	6 to 18 (10.8)	58	55	53	HIV infection was associated with excess deficit in all domains. Emotional control subscale was more affected in the HIV+ group than HIVEU. EF deficit globally bigger difference between HIV+ and HIVUU than HIVEU and HIVUUU. HIV+ more likely to have >2 subscales requiring clinical vigilance compared with PHEU and PHU.
**Judd 2016**	**Cohort**	**England**	**86% on ART**	**12 to 23**	**296**	**0**	**97**	**Class C illness had increased cognitive impairment compared with the HIV+ group, which had increased impairment compared with HIVUU group**
Nichols 2016	Cohort	United States	93% on ART	9 to 18	173	85	0	Highest levels of impairment if HIV+ with class C infection. Historical disease severity correlated with poorer performance. No consistent associations with current disease severity
Redmond 2016	Cohort	United States	93% on ART	7 to 16	212	107	0	Duration and baseline cART and history of protease inhibitor use were associated with language impairment resolution; higher viral loads before baseline associated with lower odds of resolution. No significant differences between HIV and HIVEU groups. cART use associated with lower rates of language impairment.
Brahmbhatt 2017	Cohort	Uganda	58 children on ART	7 to 14	140	26	204	Significantly more pHIV children had impairment at baseline than PHUU and PHEU. HIV-infected infants with viral suppression on ART had better recovery of developmental milestones than those without suppression, however, deficits persisted compared with uninfected infants
Malee 2017	Cohort	United States	128 children on ART	12 to 17	144	79	0	Retrospective memory poorer in children with HIV. CD4<25% at baseline predicted poorer scores
Willen 2017	Cross-sectional	United States	22 children on ART	18 to 24	29	0	13	Significant differences between groups on almost all neuropsychological variables. HIV+ youth had cognitive deficits even after adjusting for sociodemographic background
Boivin 2018	Cohort	South Africa, Zimbabwe, Malawi, & Uganda	242 children on HAART	5 to 11	246	183	182	HIV+ children performed worse than both other groups in EF, but EF improved in HIV+ children who commenced ART before 1 yr of age
**Debeaudrap 2018**	**Cross-sectional**	**Cameroon**	**All children on ART**	**4 to 9**	**127**	**101**	**110**	**Negative linear gradient in KABC-II scores from HUU to HEU to HIV+**
Harris 2018	Cohort	United States	162 children on ART	9 to 16	173	85	0	HIV+ class C disease and PHEU differed significantly on all scores, but no significant difference for children without class C disease
**Bovin 2019**	**Cohort**	**South Africa, Zimbabwe, Malawi, & Uganda**	**242 children on HAART**	**5 to 11**	**246**	**183**	**182**	**HIV+ group scored worse in tests of EF than control groups**
Kerr 2019	Cohort	Thailand & Cambodia	All children on ART	>10	231	125	138	HIV+ groups had higher odds of impaired EF, design & verbal fluency, & visual memory compared with HIVUU. HIVEU had higher odds of impaired executive function compared with HIVUU.
Van den Hof 2019 (PLoS ONE)	Cross-sectional	Netherlands	All children on ART	10	14	0	15	HIV+ scored lower on all cognitive domains, and had a significant lower IQ and a poorer cognitive profile than control group
Van den Hof 2019 (clinical Infectious diseases)	Cohort	Netherlands	All children on ART	(13.1 at first 17.7 at second)	21	0	23	Compared with HIVUU participants, in HIV+ participants the IQ score increased significantly more over time, whereas executive functioning decreased significantly more. At follow-up, the difference between groups was neutralized
Frigati 2019	Cohort	South Africa	All children on ART	9 to 14	385	0	95	HIV+ group had significantly more neurocognitive impairment than the HIVUU group
Robbins 2020	Cohort	United States	Unknown	15 to 29	206	134	0	HIV+ and HIVEU youth perform similarly on working memory and executive function domains
García-Navarro 2020	Cohort	Spain	94% on ART	Mean age 15	97	0	0	65% of cohort within average range for IQ composite score Poorer performances on “crystallized” rather than “fluid” intelligence than average
Hof 2020	Cohort	Netherlands	100% on ART	8 to 18 (13.1 at first, 17.7 at second)	21	0	23	HIV+ group scored significantly lower on composite scores than HIV− participants, with both groups scoring below the population norm. At follow-up, the difference in IQ scores between groups was neutralized, but the effect on EF remained. Lower scores for EF were seen where cART was started later
Molinaro 2021	Cohort	Zambia	100% on ART	8 to 17	208	208	0	Participants with HIV had higher levels of depressive symptoms than controls and were more likely to have cognitive impairment. Depressed mood was associated with cognitive impairment in participants with HIV but not in HEU participants
van Opstal 2021	Cross-sectional	Netherlands	100% on ART	5 to 18 (9.9)	43	24	0	More problems for HIV-infected children than their siblings
Mbewe 2022	Cohort	Zambia	100% on ART	8 to 17	208	208	0	Children with HIV performed significantly worse on a composite measure of cognitive function and were more likely to have cognitive impairment
Patil 2022	Cohort	Zambia	100% on ART	12	188	152	0	Participants with HIV scored lower on all cognitive tests than controls. Effect of HIV on cognition remained significant even excluding children with CD4<200 or WHO stage 4 disease
Arenas-Pinto 2022	Cohort	England	222 children on ART	12–21 yrs	234	0	0	Similar levels of performance in all children regardless of HIV severity
Gascon 2022	Cohort	Brazil	All children on ART	10.7	25	0	0	Mild-to-moderate cognitive alteration in 40% of the HIV+ group compared with 4.3% in sexually transmitted group

Bold print signifies a study included in meta-analysis.

#### Meta-Analysis

The 8 studies included in meta-analysis demonstrate a standardized mean difference of −0.508 (95% CI: −0.7903 to −0.2272, prediction interval −1.4609 to 0.4433) between children with HIV and their uninfected controls, indicating poorer cognitive function in HIV-infected children.

#### Synthesis Without Meta-Analysis

In total, 27 studies (60%) with control groups demonstrate that HIV-infected children perform more poorly on cognitive testing than uninfected controls (Table 3). In 2 studies, this was only in HIV-infected children who had experienced a class C event,^21,22^ but others demonstrated overall poorer cognition despite excluding children with WHO stage 4 disease or CD4 counts <200.^23^ Executive function was the most substantially affected area in 5 studies.^24–28^ Rates of asymptomatic neurocognitive impairment were higher in perinatally affected children than in older adults who had developed HIV in adulthood.^29^

Eight (17%) studies demonstrated no difference in overall cognition between HIV-infected children and their uninfected counterparts.^30–33^ However, some specific neurocognitive deficits were observed on psychometric testing.^30^ The most affected neurocognitive domains were executive function, working memory, and processing speed.

One commented that, although differences were not seen between groups, the level of cognitive impairment was much higher in both HIV-infected and HEU groups than the population average.^31^

The remaining 10 (22%) studies did not use matched control groups but compared with normative population samples and found a higher-than-expected level of schooling difficulties.^34,35^ Longer duration of ART use was associated with better performance. Again, focal cognitive deficits were seen more frequently than global deficits. Comparing HIV-positive adolescents with and without a history of class C illness found that baseline cognitive deficits were not seen at 2 years follow-up, suggesting that the adolescent brain has capacity to regenerate in the absence of further insults. Higher number of HIV-positive children required special education.

As with developmental delay, markers of early severe disease such as class C illness or lowest nadir CD4 count were consistently associated with cognitive impairment. These children performed more poorly than HEU children. The difference remained despite later immune reconstitution.^21^ The performance gap was reduced in children who had good nutrition, received early treatment with ART (usually before 1 year of age), and had never experienced a class C illness.

Every additional year of schooling was associated with decreased neurocognitive impairment in childhood and young adulthood.^36^ Improving nutritional status was associated with cognitive gain.^37^ Depression increased rates of cognitive impairment.^38^ Good adherence to ART is associated with developmental progress and this effect is most pronounced when ART is initiated early.

### Psychiatric Illness

#### Study Characteristics

Thirty-nine relevant records were identified, of which 10 contributed to the meta-analyses, including 1606 HIV-infected children and 1268 uninfected controls. Control groups consisted of children who were either exposed to HIV in utero (HIV-exposed uninfected), or who lived with a family member who had HIV (HIV affected). Studies were conducted in Brazil (1), Cameroon (1), India (1), Italy (1), Kenya (1), Poland (1), South Africa (3), Spain (1), Thailand (4), Uganda (2), United Kingdom (4), United States (17), Zambia (1), and Zimbabwe (1). Study characteristics are given in Table 4 (Fig. 2).

**TABLE 4.** Characteristics and Findings of Studies Investigating Mental Health Problems

Study	Study Design	Location	ART use	Age in years	Number of Participants			Outcomes	Prevalence/Severity of Mental Health Problems in HIV^+^ vs. Controls	Key Findings
					HIV+	HIVEU	HIVUU			
Mellins 2003	Cohort	United States	Yes	3–17	96	211	0	Emotional & behavioral symptoms	HIV^+^ = HIVEU	Higher prevalence of emotional and behavioral problems in HIV + and HIVEU groups than population norms but significant difference between groups
Malee 2011	Cohort	United States	Yes	7–16	295	121	0	Emotional & behavioral symptoms	HIV^+^ < HIVEU	Prevalence of mental health problems was higher among the PHEU than among the pHIV group (38% versus 25%, *P*= 0.01)
Smith R 2019	Cohort	United States	100%	7–16	355	196	0	Emotional & behavioral symptoms. Psychiatric diagnosis	HIV^+^ = HIVEU	Rates of mental health diagnosis and symptoms were similar between groups
**Chernoff 2009**	**Cohort**	**United States**	**Yes**	**6–17**	**319**	**174**	**84**	**Emotional & behavioral symptoms.** **Psychiatric diagnosis & treatment**	**HIV**^**+**^ **= HIVEU = HIVUU**	**High prevalence of mental health disorders in all groups of 61%, with no difference between groups. HIV+ children had twice the odds of control group of having received stimulants and more than 4 times the odds of having received antidepressants**
Marhefka 2009	Cohort	United States	Yes	13–21	99	0	0	Emotional & behavioral symptoms	N/a	31% of participants had clinically significant emotional and behavioral problems
Gadow 2010	Cohort	United States & Puerto Rico	Not stated	6–17	319	174	82	Emotional & behavioral symptoms & psychiatric diagnosis	HIV^+^ = HIVEU+HIVUU	No difference in prevalence or severity of mental health problems between groups
Gadow 2012	Cohort	United States & Puerto Rico	Yes	6–17	296	229		Emotional & behavioral symptoms & psychiatric diagnosis	HIV^+^ = HIVEU+HIVUU	Incidence rate between HIV+ group and controls was not significantly different but high overall prevalence of 70% in all groups. Asymptomatic youth with HIV were significantly more likely to receive psychotropic medication during follow-up than controls
Nachman 2012	Cohort	United States & Puerto Rico	Yes	6–17	319	0	0	Emotional & behavioral symptoms & psychiatric diagnosis	N/a	A third of participants had a psychiatric diagnosis. Lower nadir CD4 count was associated with worse cognitive functioning and social skills
Osigwe 2020	Cohort	United States	Yes	6–17	314	0	0		N/a	Higher symptom severity scores for conduct disorder in boys and girls than the normative sample and higher symptom severity for oppositional defiant disorder in boys
								Behavioral problems		
Mellins 2009	Cohort	United States	Yes	9–16	206	134	0	Emotional & behavioral symptoms & psychiatric diagnosis	HIV^+^ > HIVEU	HIV+ increased risk of psychiatric disorder (OR = 1.59; CI = 1.03,2.47; *P* < 0.05) and ADHD (OR = 2.45; CI = 1.20, 4.99, *P* < 0.05) compared with HIVEU.
**Mellins 2012**	**Cohort**	**United States**	**Yes**	**9–16**	**166**	**114**	**0**	**Emotional & behavioral symptoms & psychiatric diagnosis**	**HIV**^**+**^ **> HIVEU**	**HIV+ group was 3 times more likely to report mood disorder and 2 times more likely to report ADHD at baseline with no significant difference between groups between baseline and follow-up**
**Mutumba 2016**	**Cohort**	**United States**	**Yes**	**9–16**	**206**	**134**	**0**	**Emotional & behavioral symptoms**	**HIV**^**+**^ **= HIVEU**	**Similar levels of mental health symptoms between HIV+ and HIVEU controls**
Abrams 2018	Cohort	United States	Yes	9–12	151	97	0	Emotional & behavioral symptoms & psychiatric diagnosis	HIV^+^ = HIVEU	No significant difference in prevalence between groups, though the HIV+ group was more likely to be receiving mental health treatment
Kreniske 2019	Cohort	United States	Yes	9–16	206	134	0	Suicide attempt	HIV^+^ > HIVEU	HIV+ group was more likely to attempt suicide than control (OR 2.35, 95% CI 1.28–4.34)
Kreniske 2021	Cohort	United States	Yes	9–16	206	133	0	Suicide attempt	HIV^+^ > HIVEU	HIV+ group 2.21 times the odds of making at least 1 suicide attempt during the course of the longitudinal study compared with controls
**Le Prevost 2018**	**Cohort**	**UK**	**Yes**	**13–23**	**283**	**0**	**96**	**Emotional symptoms & self esteem**	**HIV**^**+**^ **= HIVUU**	**No significant difference between groups in anxiety & depression symptoms**
Copelyn 2019	Cohort	United Kingdom	Yes	12–21	303	0	100	Self-harm	HIV^+^ = HIVUU	HIV was not associated with self-harm in multivariable analysis compared with controls
Jantarabenjakul 2019	Cohort	Thailand	Yes	18–60 months old	41	80	0	Behavioral symptoms	HIV^+^ = HIVEU	No significant difference between groups for any behavioral symptom scoring scale
**Hoare 2019**	**Cohort**	**South Africa**	**Yes**	**9–11**	**204**	**0**	**44**	**Emotional & behavioral symptoms**	**HIV**^**+**^ **> HIVUU**	**HIV+ group had worse functional competence, self-concept and motivation, as well as higher levels of disruptive behavior, depression, and attention-deficit hyperactivity disorder symptoms and clinically significant anger and disruptive behavior than controls**
Kinyanda 2019	Cohort	Uganda	Yes	5–17	1339	0	0	Psychiatric diagnosis & symptoms	N/a	A fifth of participants had at least 1 psychiatric disorder: separation anxiety (4.6%) and ADHD (5.3%) were most common
Molinaro 2021	Cohort	Zambia	Yes	8–17	208	208	0	Depression symptoms	HIV^+^ > HIVEU	Participants with HIV demonstrated significantly higher levels of depressive symptoms than controls
Gaughan 2004	Cohort	United States	Yes	<15	1801	1021	0	Psychiatric hospitalization	HIV ≥ HIVEU	Psychiatric admissions observed in the HIV+ group but not in controls, and the HIV+ group had a significantly increased risk compared with the normative population
Rocha 2005	Cross-sectional	Brazil	Not stated	0–16	146	25	0	Psychiatric diagnosis	N/a	1 case of depression and 1 case of psychosis were identified among the HIV+ group
Mellins 2006	Cross-sectional	United States	Yes	9–16	47	0	0	Psychiatric diagnosis & symptoms	N/a	55% of participants had a psychiatric disorder and 26% had multiple disorders
Melvin 2007	Cross-sectional	United Kingdom	Yes	School age	107	0	0	Emotional & behavioral symptoms	N/a	14% had significant difficulties rated by parents
Bomba 2010	Cross-sectional	Italy	Yes	5.6–18	27	0	27	Emotional & behavioral symptoms	HIV^+^ > HIVUU	HIV+ group displayed significantly reduced psychosocial health functioning, particularly at school, compared with controls. Children with HIV-RNA above the threshold level of 50 had higher scores on the CBCL delinquent behavior
**Lee 2011**	**Cross-sectional**	**Thailand**	**Yes**	**Adolescents**	**54**	**0**	**165**	**Depression symptoms**	**HIV**^**+**^ **< HIVUU**	**HIV+ patients had lower rates on a depression inventory and the incidence of depression diagnosis was lower in this group**
**Louthrenoo 2014**	**Cross-sectional**	**Thailand**	**Yes**	**11–18**	**50**	**0**	**56**	**Emotional & behavioral symptoms**	**HIV**^**+**^ **> HIVUU**	**HIV-infected adolescents had significantly more psychosocial problems than controls**
Das 2016	Cross-sectional	India	Yes	>3	53	23	0	Emotional & behavioral symptoms	HIV^+^ > HIVEU	HIV+ group was 1.55 times significantly more likely to be suffering from psychiatric morbidity than controls
**Louw 2016**	**Cross-sectional**	**South Africa**	**Yes**	**6–16**	**78**	**0**	**30**	**Emotional & behavioral symptoms**	**HIV**^**+**^ **= HIVUU**	**Rates of emotional and behavioral problems were similar between groups**
Medin 2016	Cross-sectional	Spain	Yes	11–19	95	0	0	Emotional & behavioral symptoms	N/a	There was a risk of emotional and behavioral problems in 24.5% of participants
Zalwango 2016	Cross-sectional	Uganda	77.60%	6–18	58	56	54	Emotional & behavioral symptoms	HIV^+^ > HIVEU HIV^+^ > HIVUU	HIV+ group had significantly worse depressive and distress symptoms than HIVEU and HIVUU groups
**Abubakar 2017**	**Cross-sectional**	**Kenya**	**86%**	**12–17**	**44**	**53**	**33**	**Depression symptoms**	**HIV**^**+**^ **= HIVEU HIV**^**+**^ **> HIVUU HIV**^**+**^ **> HIVEU**	**Depression symptom scores were significantly worse and there was a higher psychosocial risk for HIV+ and HIVEU groups than controls, with no significant difference between HIV+ and HIVEU groups**
Woollett 2017	Cross-sectional	South Africa	Yes	13–19	343	0	0	Anxiety, depression, PTSD, & suicidality symptoms	N/a	27% of participants had depression, anxiety, or PTSD, and 24% reported suicidality. Peer violence was correlated with all health problems
**Rukuni 2018**	**Cross-sectional**	**Zimbabwe**	**100%**	**6–16**	**202**	**0**	**285**	**Emotional symptoms**	**HIV**^**+**^ **> HIVUU**	**HIV-infected children were significantly more likely to report anxiety (aOR 4.4 95% CI 2.4, 8.1) and low mood (aOR 4.2 95% CI 2.1, 8.4)**
Zielinska-Wieniawska 2021	Cross-sectional	Poland	87.50%	6-18y/o	56	24	43	Emotional & behavioral symptoms	HIV^+^ ≥ HIVUU HIV+ ≥ HIVEU	The prevalence of psychiatric disorders was not significantly different between groups, but HIV+ group had worse symptoms in some subsets. Psychiatric disorders more common among HIV+ children where ART was started after 12 months old. HIV severity before treatment was associated with severity of internalizing problems
Maliik 2022	Cross-sectional	United Kingdom	Yes	>18y/o	184	0	0	Psychosis	N/a	The prevalence of psychosis in participants was above the lifetime prevalence of psychosis in UK individuals aged 16–34 years
Ndongo 2023	Cross-sectional	Cameroon	100%	10-19y/o	302	0	0	Psychiatric symptoms	N/a	This study found prevalence of 26.5% for severe depression, 36.4% for suicidal ideation, 29.1% for high/very high anxiety, and 20.5% for low self-esteem
Chaiudosom 2022	Cross-sectional	Thailand	100%	12-24y/o	79	0	0	Depression diagnosis	N/a	The prevalence of depressive disorders in adolescents with perinatally acquired HIV was 17.7%. Depressive disorders were found to be one of the factors associated with virologic failure

Bold signifies studies included in meta-analysis.

#### Meta-Analysis

**FIGURE 3.**
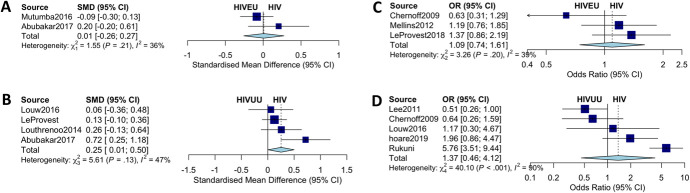
Anxiety and depression in HIV-infected verses uninfected children. A, Continuous measures of depression and anxiety symptom scores in HIV+ and HIVEU children. B, Anxiety and depression symptom scores in HIV+ and HIVUU children. (C) Diagnoses of depression/anxiety in HIV+ and HIVEU children. (D) Diagnosis of depression/anxiety in HIV+ and HIVUU children.

Studies that reported symptom scores for depression and anxiety or gave a diagnosis of depression or anxiety contributed to these meta-analyses. We did not find evidence of a difference of diagnosis of depression or anxiety in children with HIV (odds ratio 1.105 (CI: 0.778 to 1.571; prediction intervals 0.0505; 24.1953)) than their exposed uninfected controls. We did not find evidence of a difference in terms of anxiety and depression symptom scores between HIV-infected children and their exposed uninfected controls (SMD 0.0096; 95% CI: −0.2585 to 0.2777]). We also did not find evidence of a difference in symptom score (SMD 1.3868; 95% CI: 0.5473 to 3.5138) or diagnosis rate (SMD 0.2559; 95% CI: 0.0054 to 0.5064) of anxiety and depression between children with HIV and unexposed uninfected controls (Fig. 3).

#### Synthesis Without Meta-Analysis

The most notable finding is that studies report very high levels of psychiatric impairment in both HIV-infected and HIV-affected (control) groups of children. Studies without control groups observed high rates of psychiatric illness in children with HIV (55% in 1 study), including a higher than population average rate of psychosis. Disorders reported on include depression, anxiety, self-harm, suicidality, ADHD, oppositional defiant disorder, conduct disorder, and psychosis. In total, 11 studies report a greater degree of psychiatric impairment in children with HIV than HIVEU/HIV unexposed controls, 13 studies did not find a difference between groups and 2 studies report a greater degree of psychiatric impairment in children who were exposed but uninfected than children who were HIV positive (see Table 4). Out of 23 studies that could not contribute to meta-analysis, 8 studies with control groups reported higher incidence of mental health problems in children with perinatally acquired HIV than their counterparts. There were some other specific mental health difficulties reported by studies that could not contribute to meta-analysis. Rates of ADHD were higher in children with HIV than in their HIV-exposed, uninfected controls, as were undiagnosed attention and concentration difficulties.^39–41^ One study reported higher risk of conduct disorder and oppositional defiant disorder in children with HIV (Table 4). Another reported that children with HIV were more likely to have received both stimulants and antidepressants than control children. Two studies reported higher risk of suicide attempt in children with HIV than in the control group.

There was not a clear association between HIV severity status, nadir CD4 count or ART use, and psychiatric diagnosis or severity with mixed results from studies.

Studies without control groups reported high levels (between 27% and 70%) of mental health disturbance in both HIV-positive children and HIV-exposed, uninfected controls, or children growing up in families affected by HIV^42^ (see Table 4). Anxiety, depression, and “behavioral” disorders were the most common diagnoses. A high proportion reported suicidality.^43^

### Young People's Advisory Group

Detailed information about the Young People's advisory group can be found in the appendix. The purpose of the conversation was to elicit what young people would like to know about possible neurologic and psychiatric effects of perinatally acquired HIV, and how any information could be usefully shared, and whom with. The group advised us that the main issues remain stigma and the impact on medication adherence. They thought this was the main stressor affecting their mental health. Most young people had not disclosed their HIV status to their teacher. Guardians commented that knowing early about possible cognitive effects may allow them to spot early signs and seek timely help.

## DISCUSSION

In this systematic review and meta-analysis, we found that there is a burden of neuropsychiatric impairment in children with pediatric HIV that is emerging with improved life expectancy. Although this seems to have improved with early initiation and adherence to ART, there is evidence of some deficits despite optimal treatment.

Impairment becomes apparent in early childhood, when children with pHIV are more likely to experience developmental delay than their uninfected peers. Although social factors, such as parental loss, poverty, and nutrition, contribute, the association persists even when compared with children with HIVEU. This suggests that HIV infection itself might play a role in contributing to neurodevelopmental delay. This is further supported by the fact that early use of cART and lower burden of HIV in early life are associated with better neurodevelopment. Early intervention, ideally between conception and 2 years of age, is likely to be critical in mitigating developmental delay.^44^

Although early use of cART is part of the answer, it is not a complete solution, as globally children face several barriers to adherence, including availability, side effects, social stigma, and difficult multidrug regimes. Although most children in the later studies that we included (Tables 2–4) were on ART, UNICEF data report that in 2023 only 57% of children with HIV globally were receiving ART. In addition, our findings show that some neurodevelopmental impairment remains despite optimal use of cART and in the absence of class C illness (illnesses that, when contracted in a child with HIV, define progression to AIDS). HIV is adept at crossing the blood–brain barrier, but most forms of ART are not.^45^

Consequently, as the CNS is recognized as a potential reservoir for HIV, this complicates efforts to eradicate the virus completely. In adults, neurologic sequelae, such as HIV-associated dementia, have long been documented, underscoring the significant impact of CNS involvement in the disease and its progression. In addition, there are some concerns that ART with enhanced neuropenetrative effects may inadvertently introduce neurotoxic side effects—an issue of particular concern in the pediatric population.^46^ Given the current limitations in eradicating CNS HIV, there is a need to develop nonpharmacologic strategies (such as parent led developmental interventions) to mitigate neurocognitive sequelae and further investigate the underlying mechanisms to better support younger patients.

Evidence for developmental interventions in perinatally HIV-infected children is lacking,^47^ although they have proven useful in other vulnerable populations, such as children born prematurely. Our data demonstrate a scope of developmental differences meaning that such interventions are likely to be valuable for children with pHIV.^48^

Cognitive difficulties can persist throughout schooling and into young adulthood. As for developmental delay, we observed an effect of HIV on cognition, independent of class C illness and socioeconomic background. Executive function and memory were specific areas of cognitive difficulty. This marries neuroimaging data demonstrating structural and functional changes in the brains of infected children, which were present despite adequate viral suppression.^49,50^ Differences on cognitive rating scores in some studies were supported by other studies reporting higher number of HIV-positive children requiring special education or being in age-discordant school year groups, demonstrating a level of functional impairment. It is important to note that these difficulties persist despite early use of ART and, therefore, that other interventions are required. However, we have also noted that there is a large real-world population of children who do not receive optimal ART in a timely fashion, who may benefit from targeted treatments for these cognitive difficulties to support them with their education.

Although we report a direct effect of HIV on the developing brain, children growing up with HIV also are affected by social factors that leave them vulnerable to neurocognitive and psychiatric difficulties. Children with a HIV-positive mother are more likely to leave education earlier than children without, regardless of HIV status of the child. Each additional year of schooling in children with HIV is associated with improved cognitive performance.^36,51^ In fact, children with pHIV are more likely to miss school or to drop out of school altogether, limiting the opportunities for optimizing their neurodevelopment.^15^ Parental loss and unemployment were also independently associated with cognitive impairment, and malnutrition was a strong predictive factor for cognitive impairment in several studies.^37^ Emotional and behavioral difficulties of children were also associated with their cognitive outcome. Addressing cognitive difficulties in HIV-affected children, therefore, requires a multidisciplinary approach: adapting ART for better CNS penetrance and less neurotoxicity, improving school experiences and retainment, avoiding malnutrition, promoting good mental health, and developing interventions that support early development.

Our findings for mental health outcomes are less clear because studies do not agree whether HIV causes an effect on mental health independently of the social factors from growing up in a household affected by HIV. Studies were predominantly conducted in high-income countries among young people engaged in treatment programs. However, we know that worldwide only 57% of children with pHIV are on ART.^4^

We did not find evidence of difference between children with or without HIV in terms of receiving a diagnosis of anxiety or depression. Narrative synthesis showed variable effects of HIV, but symptom prevalence was often high in both affected and exposed-uninfected counterparts. The lack of difference may mean that the psychiatric sequelae of pediatric HIV are environmental, but it is also important to note that the population used for the mental health cohorts is not completely representative of the average child with HIV. Nevertheless, there is clearly a large burden of impairment in this population warranting treatment. Further cohort studies for a longer follow-up period in low–middle-income settings are needed.

This analysis highlights the potential synergistic effect of mental health problems on cognition and development. Depressed mood is more prevalent in HIV-affected populations (including children infected with HIV, children perinatally exposed to HIV but uninfected, and children living in affected families) and independently affects cognition. Attention and concentration difficulties are more common in children with HIV, and these can limit access to and performance in education.

This review shows an association between growing up with HIV and poorer CNS outcomes. Although it is well established that HIV has a direct effect on the developing brain, this is only partially alleviated by current cART regimes. In addition, real-world rates of compliance with ART are reported to be lower than seen in these studies,^4^ highlighting both the need to address chronic complications of pediatric HIV and to design studies that better capture the experience of the typical child with perinatally acquired HIV. Increasing ART distribution and compliance, adapting ART for CNS penetrance, and introducing interventions to support cognitive development could help. We also identified several indirect factors associated with the family environment, such as malnutrition or parental loss and unemployment: intervention that is available for whole families may reduce the role of these factors in neuropsychiatric impairment.

### Patient and Public Involvement

We held a focus group discussion on January 20, 2022 with young people with pHIV to better understand how our findings could help. The predominant concern of the young people was social stigma generally but particularly at school with their teachers. Their main desire was to improve public education around HIV to reduce social stigma, which, in turn, would improve their ability to access support. Difficulties in disclosing HIV status to teachers may have limited support that the young people may have recieved with cognitive and developmental problems they were facing. They suggested that posters at bus stops, leaflets at schools, and programs on the local radio may be a useful way to disseminate this information.

### Limitations

Our study had several strengths. As the inclusion criteria were broad, this review allows a much broader oversight of the neurologic and psychiatric consequences of perinatally acquired HIV than other similar studies. Second, we investigated several outcomes related to early neurodevelopment, which allows insights into different types of neuropsychiatric impairment. The narrative synthesis provides an overview of the whole scope of neuropsychiatric impairment in children with perinatally acquired HIV over time.

However, there are also some important limitations. In most cases, studies included in the meta-analysis described differences in cognition/development using standardized scoring scales. This is useful in that it is validated, repeatable, and can be used in a wide range of contexts without depending on what task the individual is trying to achieve. However, when addressing the individual patient, measures of functional impairment are more useful—what goals the person would like to achieve that they are unable to because of their cognition. Because functional impairment depends on the context, it is difficult to say which level of “reduction” on a test score leads to clinically meaningful impairment. Some studies use functional data (i.e. being in an age-discordant school year/needing special education) and this helps to demonstrate that there is some functional impairment, although a limitation is that it cannot be exactly quantified.

The population of children included were largely children already engaged in medical programs for HIV and were receiving treatment. These children are not likely to be completely representative of the true population of children with HIV. In total, 80% of children with pHIV live in sub-Saharan Africa, but this is not reflected in the population in the studies. A large proportion of the studies included in the meta-analysis for developmental delay were conducted in the pre-ART era and, therefore, may be less applicable to children affected with HIV today. However, UNICEF data suggest that real-world access and adherence to ART remain lower than that reported in more recent studies (see Tables 2–4),^4^ and, therefore, these data may still be of relevance to current populations. Owing to concerns about finding false positive findings from multiple analyses, we have not, in this study, conducted additional comparisons of neuropsychiatric impairment between countries, year, and socioeconomic context. This could be a potential area for further research.

The overall proportion of studies that were included in the meta-analysis was low. This is because some studies not having directly comparable outcome measures, missing data, and different, or lack of, control groups in some studies. Different control groups were used, with some studies using HIV-exposed, uninfected children and others using children living in HIV-affected households. In the meta-analysis, sometimes these different control groups were pooled, as our aim was to investigate the neuropsychiatric disorders affecting children infected with HIV. However, because children who are exposed but uninfected with HIV have another mechanism of HIV-induced developmental delay, they may be a less-than-ideal control group. This is partially because of different ART regimes used between studies, reflecting the time course over which they were carried out. Although the quality of studies, Supplemental Digital Content (see http://links.lww.com/QAI/C418) was generally “good” or “adequate,” attention to specific aspects of the Newcastle-Ottawa scoring system highlights that no studies used masking when assessing for neuropsychiatric impairment and that often the control group was recruited separately from the infected group, raising the risk of bias. Given this heterogeneity, the results of meta-analysis should be interpreted with caution, but they nonetheless provide a useful indication to those working with children with pHIV on the neurodevelopmental impairment that they are likely to face.

## CONCLUSIONS

The story of HIV has been almost entirely rewritten for the past 3 decades with the advent and rollout of ART. No longer leading to death in infancy, it has become a chronic disease to be managed throughout life. Many of the sequelae of perinatally acquired HIV affect the central nervous system, possibly because of direct viral effects, as well as inadequate penetrance of ART across the blood–brain barrier and indirect effects of living with HIV. Despite decades of research into children with perinatally acquired HIV, there are limited programs aimed at improving their neurodevelopmental outcomes. Early and effective ART is essential to optimize neurologic outcomes for this group, but it is also clear that good nutrition, developmental support, and social interventions are necessary. To meet the needs of this population fully, services must account for the spectrum of HIV-related neurocognitive impairment and mental illness. Opportunities remain to develop interventions aimed at reducing neurologic impairments and include both redesign of ART with consideration of the blood–brain barrier and development of interventions to support cognition and mental health in affected children across home, community, and educational settings.

## Supplementary Material
