# Supplementary material for: Neuropsychiatric Outcomes in Children and Adolescents With Perinatally Acquired HIV: A Systematic Review and Meta-Analysis
Source: J Acquir Immune Defic Syndr. 2025 Mar 10;98(5):411–28. doi: 10.1097/QAI.0000000000003595 (PMC11893004; doi:10.1097/QAI.0000000000003595)
Supplement: Supplementary file 1 [file qai-98-411-s001.docx]

Supplementary Information

## S1- Literature search methods

*Search*

Nine databases were searched on 30/05/2023 by an information specialist (EH): MEDLINE, Embase, and PsycINFO (all via Ovid SP); CINAHL and Child Development and Adolescent Studies (via EBSCO); the Web of Science Core Collection; Scopus; ProQuest Dissertations and Theses Global; and WHO Global Index Medicus. No limits were applied. Search strategies incorporated keywords and thesaurus headings to describe children and adolescents aged 0-25 with perinatally-acquired HIV and terms to describe the spectrum of neuropsychiatric impairment. See PROSPERO registration CRD42020159159.  Results were deduplicated manually using EndNote 20.

*Data Extraction*

Papers titles were screened first by two independent reviewers (RH and AM), following which abstracts from eligible titles were further screened). Finally, the two reviewers (RH and AM) assessed eligible full texts for inclusion against the pre-defined inclusion/exclusion criteria. Data were extracted into Microsoft Excel and double checked by an independent data extractor. Data extracted included study type, sample strategy, sample size, measurement method with means and standard deviations in scores where available.

*Study Appraisal*

R.H and A.M independently appraised papers using the Newcastle-Ottawa Scoring System (see below). Disagreement was settled with discussion.

## Search Strategies

*Database:* **Medline (Ovid MEDLINE® Epub Ahead of Print, In-Process & Other Non-Indexed Citations, Ovid MEDLINE® Daily and Ovid MEDLINE®) 1946 to present**

<https://ovidsp.ovid.com/ovidweb.cgi?T=JS&NEWS=N&PAGE=main&SHAREDSEARCHID=3RMMSemSdLVHSt25KilEI1P02m1ZlDebwb5Rz0MYDNtlIkLhf0vRNOqc02EgvIhUE>

*Search Strategy:***1**  (infan* or newborn* or new-born* or perinat* or neonat* or baby* or babies or toddler* or minor* or boy* or girl* or kid or kids or child* or schoolchild* or adolescen* or juvenil* or youth* or teen* or pubescen* or pediatric* or paediatric* or peadiatric* or school* or prematur* or preterm* or "pre term*" or prepub* or pre-pub* or preschool* or pre-school* or kindergarten* or nursery* or preadolescen* or pre-adolescen*).tw. (3437307)
**2**  adolescent/ or exp child/ or exp infant/ (3953015)
**3**  ("young adult*" or "young person*" or "young people" or "young woman" or "young women" or "young man" or "young men").tw. (206912)
**4**  Young Adult/ (1009447)
**5**  1 or 2 or 3 or 4 (5808296)
**6**  ("Psychiatric Disorder*" or "Mental Disorder*" or Anxiety or Anxious or Phobia* or Phobic or Neurotic or Neurosis or "conversion disorder" or "medically unexplained symptoms" or PTSD or "Post Traumatic Stress Disorder" or Depress*).tw. (774470)
**7**  (Psychosis or Psychotic or "Cognition dysfunction" or "Cognitive function" or memory or "cognitive disorder" or "cognitive deficit" or "Executive function" or "Neuropsychological test*" or "Brain Disorder*" or "Mood Disorder*" or "Affective disorder*" or Epilepsy or Epileptic or "Emotional Disorder").tw. (591514)
**8**  (Schizopreni* or Autism or ASD or Autistic or "Bipolar Disorder*" or "Mental* Ill*" or "Neurocognitive Disorder*" or "Neurodevelopmental Disorder*" or "Substance Abuse" or "Substance Misuse" or ADHD or "Attention deficit hyperactivity disorder" or "Behaviour* Disorder*" or "Behavior disorder").tw. (224384)
**9**  ("Emotional Disturbance*" or Suicid* or "Self Harm" or "Self Injur*" or "Self Destructive Behaviour" or "Self Destructive Behavior" or OCD or "Obsessive Compulsive Disorder" or "Neurological Disorder*" or "Neurological Disease*" or "Alcohol Misuse" or "Alcohol Abuse" or "Alcoholic" or "Conduct Disorder*" or "Oppositional Defiant Disorder" or paranoia or "hearing voices" or hallucination* or mania or manic or "eating disorder*" or "anorexia nervosa" or bulimi* or "binge eat*").tw. (334380)
**10**  exp Mental Disorders/ (1427327)
**11**  Mental Health/ (60598)
**12**  6 or 7 or 8 or 9 or 10 or 11 (2512382)
**13**  Infectious Disease Transmission, Vertical/ (18721)
**14**  (phiv or (vertical* adj4 transmi*)).tw. (10823)
**15**  (perinatal* adj4 (infect* or Acquir* or transmi* or expos*)).tw. (10344)
**16**  (peri-natal* adj4 (infect* or Acquir* or transmi* or expos*)).tw. (21)
**17**  (Fetomaternal* adj4 (infect* or Acquir* or transmi* or expos*)).tw. (31)
**18**  ("mother to child" adj4 (infect* or Acquir* or transmi* or expos*)).tw. (7332)
**19**  ("maternal fetal" adj4 (infect* or Acquir* or transmi* or expos*)).tw. (731)
**20**  (maternofetal adj4 (infect* or Acquir* or transmi* or expos*)).tw. (138)
**21**  13 or 14 or 15 or 16 or 17 or 18 or 19 or 20 (36016)
**22**  exp HIV/ (107311)
**23**  (hiv or "human immunodeficiency virus*" or hiv-1 or hiv-2 or "acquired immune deficiency syndrome*" or "human immune-deficiency virus*" or "acquired immunodeficiency syndrome*" or AIDS or "acquired immuno-deficiency syndrome*").tw. (456138)
**24**  22 or 23 (461704)
**25**  5 and 12 and 21 and 24 (911)

*Database***: Embase 1974 to present**

<https://ovidsp.ovid.com/ovidweb.cgi?T=JS&NEWS=N&PAGE=main&SHAREDSEARCHID=4dhrJl6eM85tY60UNasfIzEl5zDWibuDUBcxrLPW06zJFNpATrbJ4Wp7xrzi9m1NJ>

**1**  (infan* or newborn* or new-born* or perinat* or neonat* or baby* or babies or toddler* or minor* or boy* or girl* or kid or kids or child* or schoolchild* or adolescen* or juvenil* or youth* or teen* or pubescen* or pediatric* or paediatric* or peadiatric* or school* or prematur* or preterm* or "pre term*" or prepub* or pre-pub* or preschool* or pre-school* or kindergarten* or nursery* or preadolescen* or pre-adolescen*).tw. (4360852)
**2**  adolescent/ or exp child/ or exp infant/ (3940544)
**3**  ("young adult*" or "young person*" or "young people" or "young woman" or "young women" or "young man" or "young men").tw. (278229)
**4**  Young Adult/ (507976)
**5**  1 or 2 or 3 or 4 (6072642)
**6**  ("Psychiatric Disorder*" or "Mental Disorder*" or Anxiety or Anxious or Phobia* or Phobic or Neurotic or Neurosis or "conversion disorder" or "medically unexplained symptoms" or PTSD or "Post Traumatic Stress Disorder" or Depress*).tw. (1046149)
**7**  (Psychosis or Psychotic or "Cognition dysfunction" or "Cognitive function" or memory or "cognitive disorder" or "cognitive deficit" or "Executive function" or "Neuropsychological test*" or "Brain Disorder*" or "Mood Disorder*" or "Affective disorder*" or Epilepsy or Epileptic or "Emotional Disorder").tw. (811307)
**8**  (Schizopreni* or Autism or ASD or Autistic or "Bipolar Disorder*" or "Mental* Ill*" or "Neurocognitive Disorder*" or "Neurodevelopmental Disorder*" or "Substance Abuse" or "Substance Misuse" or ADHD or "Attention deficit hyperactivity disorder" or "Behaviour* Disorder*" or "Behavior disorder").tw. (312085)
**9**  ("Emotional Disturbance*" or Suicid* or "Self Harm" or "Self Injur*" or "Self Destructive Behaviour" or "Self Destructive Behavior" or OCD or "Obsessive Compulsive Disorder" or "Neurological Disorder*" or "Neurological Disease*" or "Alcohol Misuse" or "Alcohol Abuse" or "Alcoholic" or "Conduct Disorder*" or "Oppositional Defiant Disorder" or paranoia or "hearing voices" or hallucination* or mania or manic or "eating disorder*" or "anorexia nervosa" or bulimi* or "binge eat*").tw. (466087)
**10**  exp mental disease/ (2624878)
**11**  exp mental health/ (231705)
**12**  6 or 7 or 8 or 9 or 10 or 11 (3724297)
**13**  vertical transmission/ (18012)
**14**  (phiv or (vertical* adj4 transmi*)).tw. (13456)
**15**  (perinatal* adj4 (infect* or Acquir* or transmi* or expos*)).tw. (13507)
**16**  (peri-natal* adj4 (infect* or Acquir* or transmi* or expos*)).tw. (36)
**17**  (Fetomaternal* adj4 (infect* or Acquir* or transmi* or expos*)).tw. (44)
**18**  ("mother to child" adj4 (infect* or Acquir* or transmi* or expos*)).tw. (9267)
**19**  ("maternal fetal" adj4 (infect* or Acquir* or transmi* or expos*)).tw. (907)
**20**  (maternofetal adj4 (infect* or Acquir* or transmi* or expos*)).tw. (179)
**21**  13 or 14 or 15 or 16 or 17 or 18 or 19 or 20 (41916)
**22**  exp Human immunodeficiency virus/ (223916)
**23**  (hiv or "human immunodeficiency virus*" or hiv-1 or hiv-2 or "acquired immune deficiency syndrome*" or "human immune-deficiency virus*" or "acquired immunodeficiency syndrome*" or AIDS or "acquired immuno-deficiency syndrome*").tw. (586099)
**24**  22 or 23 (607328)
**25**  5 and 12 and 21 and 24 (1127)

*Database:* **PsycINFO 1806 to present**

<https://ovidsp.ovid.com/ovidweb.cgi?T=JS&NEWS=N&PAGE=main&SHAREDSEARCHID=3ToIl8694A3GXO0tSvI2toDOAvxVrbr3LVs5aawgnJDb3x2PrQtRi7O1mRohfghgW>

Search Strategy:

**1**  (infan* or newborn* or new-born* or perinat* or neonat* or baby* or babies or toddler* or minor* or boy* or girl* or kid or kids or child* or schoolchild* or adolescen* or juvenil* or youth* or teen* or pubescen* or pediatric* or paediatric* or peadiatric* or school* or prematur* or preterm* or "pre term*" or prepub* or pre-pub* or preschool* or pre-school* or kindergarten* or nursery* or preadolescen* or pre-adolescen*).tw. (1441097)
**2**  ("young adult*" or "young person*" or "young people" or "young woman" or "young women" or "young man" or "young men").tw. (110224)
**3**  1 or 2 (1486849)
**4**  ("Psychiatric Disorder*" or "Mental Disorder*" or Anxiety or Anxious or Phobia* or Phobic or Neurotic or Neurosis or "conversion disorder" or "medically unexplained symptoms" or PTSD or "Post Traumatic Stress Disorder" or Depress*).tw. (615395)
**5**  (Psychosis or Psychotic or "Cognition dysfunction" or "Cognitive function" or memory or "cognitive disorder" or "cognitive deficit" or "Executive function" or "Neuropsychological test*" or "Brain Disorder*" or "Mood Disorder*" or "Affective disorder*" or Epilepsy or Epileptic or "Emotional Disorder").tw. (418102)
**6**  (Schizopreni* or Autism or ASD or Autistic or "Bipolar Disorder*" or "Mental* Ill*" or "Neurocognitive Disorder*" or "Neurodevelopmental Disorder*" or "Substance Abuse" or "Substance Misuse" or ADHD or "Attention deficit hyperactivity disorder" or "Behaviour* Disorder*" or "Behavior disorder").tw. (233037)
**7**  ("Emotional Disturbance*" or Suicid* or "Self Harm" or "Self Injur*" or "Self Destructive Behaviour" or "Self Destructive Behavior" or OCD or "Obsessive Compulsive Disorder" or "Neurological Disorder*" or "Neurological Disease*" or "Alcohol Misuse" or "Alcohol Abuse" or "Alcoholic" or "Conduct Disorder*" or "Oppositional Defiant Disorder" or paranoia or "hearing voices" or hallucination* or mania or manic or "eating disorder*" or "anorexia nervosa" or bulimi* or "binge eat*").tw. (232106)
**8**  exp Mental Disorders/ (1022967)
**9**  Mental Health/ (88092)
**10**  4 or 5 or 6 or 7 or 8 or 9 (1644746)
**11**  (phiv or (vertical* adj4 transmi*)).tw. (523)
**12**  (perinatal* adj4 (infect* or Acquir* or transmi* or expos*)).tw. (1568)
**13**  (peri-natal* adj4 (infect* or Acquir* or transmi* or expos*)).tw. (3)
**14**  (Fetomaternal* adj4 (infect* or Acquir* or transmi* or expos*)).tw. (2)
**15**  ("mother to child" adj4 (infect* or Acquir* or transmi* or expos*)).tw. (895)
**16**  ("maternal fetal" adj4 (infect* or Acquir* or transmi* or expos*)).tw. (21)
**17**  (maternofetal adj4 (infect* or Acquir* or transmi* or expos*)).tw. (2)
**18**  11 or 12 or 13 or 14 or 15 or 16 or 17 (2775)
**19**  exp HIV/ (47296)
**20**  (hiv or "human immunodeficiency virus*" or hiv-1 or hiv-2 or "acquired immune deficiency syndrome*" or "human immune-deficiency virus*" or "acquired immunodeficiency syndrome*" or AIDS or "acquired immuno-deficiency syndrome*").tw. (78615)
**21**  19 or 20 (78787)
**22**  3 and 10 and 18 and 21 (238)

**EBSCO CINAHL** Top of Form

# EBSCOhost CINAHL

| **#** | **Query** | **Results** |
| --- | --- | --- |
| S1 | TX infan* or newborn* or new-born* or perinat* or neonat* or baby* or babies or toddler* or minor* or boy* or girl* or kid or kids or child* or schoolchild* or adolescen* or juvenil* or youth* or teen* or pubescen* or pediatric* or paediatric* or peadiatric* or school* or prematur* or preterm* or "pre term*" or prepub* or pre-pub* or preschool* or pre-school* or kindergarten* or nursery* or preadolescen* or pre-adolescen* | 1,705,631 |
| S2 | (MH "Child+") OR (MH "Young Adult") OR (MH "Adolescence+") | 1,216,671 |
| S3 | TX ("young adult*" or "young person*" or "young people" or "young woman" or "young women" or "young man" or "young men") | 350,209 |
| S4 | S1 OR S2 OR S3 | 1,836,489 |
| S5 | TX ("Psychiatric Disorder*" or "Mental Disorder*" or Anxiety or Anxious or Phobia* or Phobic or Neurotic or Neurosis or "conversion disorder" or "medically unexplained symptoms" or PTSD or "Post Traumatic Stress Disorder" or Depress*) | 385,461 |
| S6 | TX (Psychosis or Psychotic or "Cognition dysfunction" or "Cognitive function" or memory or "cognitive disorder" or "cognitive deficit" or "Executive function" or "Neuropsychological test*" or "Brain Disorder*" or "Mood Disorder*" or "Affective disorder*" OR Epilepsy or Epileptic or "Emotional Disorder") | 207,880 |
| S7 | TX (Schizopreni* or Autism or ASD or Autistic or "Bipolar Disorder*" or "Mental* Ill*" or "Neurocognitive Disorder*" or "Neurodevelopmental Disorder*" or "Substance Abuse" or "Substance Misuse" or ADHD or "Attention deficit hyperactivity disorder" or "Behaviour* Disorder*" or "Behavior disorder") | 0 |
| S8 | TX ("Emotional Disturbance*" or Suicid* or "Self Harm" or "Self Injur*" or "Self Destructive Behaviour" or "Self Destructive Behavior" or OCD or "Obsessive Compulsive Disorder" or "Neurological Disorder*" or "Neurological Disease*" or "Alcohol Misuse" or "Alcohol Abuse" or "Alcoholic" or "Conduct Disorder*" or "Oppositional Defiant Disorder" or paranoia or "hearing voices" or hallucination* or mania or manic or "eating disorder*" or "anorexia nervosa" or bulimi* or "binge eat*") | 152,046 |
| S9 | (MH "Mental Disorders+") | 639,826 |
| S10 | (MH "Mental Health") | 54,304 |
| S11 | S5 OR S6 OR S7 OR S8 OR S9 OR S10 | 1,014,754 |
| S12 | (MH "Disease Transmission, Vertical") | 6,838 |
| S13 | TX phiv or (vertical* n4 transmi*) | 7,625 |
| S14 | TX perinatal* n4 (infect* or Acquir* or transmi* or expos*) | 2,946 |
| S15 | TX peri-natal* n4 (infect* or Acquir* or transmi* or expos*) | 4 |
| S16 | TX Fetomaternal* n4 (infect* or Acquir* or transmi* or expos*) | 10 |
| S17 | TX ("mother to child" n4 (infect* or Acquir* or transmi* or expos*)) | 2,534 |
| S18 | TX ("maternal fetal" n4 (infect* or Acquir* or transmi* or expos*)) | 168 |
| S19 | TX (maternofetal n4 (infect* or Acquir* or transmi* or expos*)) | 21 |
| S20 | S12 OR S13 OR S14 OR S15 OR S16 OR S17 OR S18 OR S19 | 10,475 |
| S21 | (MH "Human Immunodeficiency Virus+") | 11,171 |
| S22 | TX hiv or "human immunodeficiency virus*" or hiv-1 or hiv-2 or "acquired immune deficiency syndrome*" or "human immune-deficiency virus*" or "acquired immunodeficiency syndrome*" or AIDS or "acquired immuno-deficiency syndrome*" | 176,607 |
| S23 | S21 OR S22 | 176,607 |
| S24 | S4 AND S11 AND S20 AND S23 | 515 |

Bottom of Form

| **#** | **Query** | **Results** |
| --- | --- | --- |
| S1 | TX infan* or newborn* or new-born* or perinat* or neonat* or baby* or babies or toddler* or minor* or boy* or girl* or kid or kids or child* or schoolchild* or adolescen* or juvenil* or youth* or teen* or pubescen* or pediatric* or paediatric* or peadiatric* or school* or prematur* or preterm* or "pre term*" or prepub* or pre-pub* or preschool* or pre-school* or kindergarten* or nursery* or preadolescen* or pre-adolescen* | 367,336 |
| S2 | TX "young adult*" or "young person*" or "young people" or "young woman" or "young women" or "young man" or "young men" | 22,419 |
| S3 | S1 OR S2 | 371,449 |
| S4 | TX "Psychiatric Disorder*" or "Mental Disorder*" or Anxiety or Anxious or Phobia* or Phobic or Neurotic or Neurosis or "conversion disorder" or "medically unexplained symptoms" or PTSD or "Post Traumatic Stress Disorder" or Depress* | 38,164 |
| S5 | TX Psychosis or Psychotic or "Cognition dysfunction" or "Cognitive function" or memory or "cognitive disorder" or "cognitive deficit" or "Executive function" or "Neuropsychological test*" or "Brain Disorder*" or "Mood Disorder*" or "Affective disorder*" OR Epilepsy or Epileptic or "Emotional Disorder" | 18,086 |
| S6 | TX Schizopreni* or Autism or ASD or Autistic or "Bipolar Disorder*" or "Mental* Ill*" or "Neurocognitive Disorder*" or "Neurodevelopmental Disorder*" or "Substance Abuse" or "Substance Misuse" or ADHD or "Attention deficit hyperactivity disorder" or "Behaviour* Disorder*" or "Behavior disorder" | 28,292 |
| S7 | TX "Emotional Disturbance*" or Suicid* or "Self Harm" or "Self Injur*" or "Self Destructive Behaviour" or "Self Destructive Behavior" or OCD or "Obsessive Compulsive Disorder" or "Neurological Disorder*" or "Neurological Disease*" or "Alcohol Misuse" or "Alcohol Abuse" or "Alcoholic" or "Conduct Disorder*" or "Oppositional Defiant Disorder" or paranoia or "hearing voices" or hallucination* or mania or manic or "eating disorder*" or "anorexia nervosa" or bulimi* or "binge eat*" | 16,813 |
| S8 | TX "mental health" | 30,433 |
| S9 | S4 OR S5 OR S6 OR S7 OR S8 | 103,239 |
| S10 | TX phiv or (vertical* n4 transmi*) | 51 |
| S11 | TX perinatal* n4 (infect* or Acquir* or transmi* or expos*) | 166 |
| S12 | TX peri-natal* n4 (infect* or Acquir* or transmi* or expos*) | 1 |
| S13 | TX Fetomaternal* n4 (infect* or Acquir* or transmi* or expos*) | 0 |
| S14 | TX "mother to child" n4 (infect* or Acquir* or transmi* or expos*) | 83 |
| S15 | TX "maternal foetal" n4 (infect* or Acquir* or transmi* or expos*) | 4 |
| S16 | TX "maternal fetal" n4 (infect* or Acquir* or transmi* or expos*) | 1 |
| S17 | TX maternofetal n4 (infect* or Acquir* or transmi* or expos*) | 2 |
| S18 | S10 OR S11 OR S12 OR S13 OR S14 OR S15 OR S16 OR S17 | 284 |
| S19 | TX hiv or "human immunodeficiency virus*" or hiv-1 or hiv-2 or "acquired immune deficiency syndrome*" or "human immune-deficiency virus*" or "acquired immunodeficiency syndrome*" or AIDS or "acquired immuno-deficiency syndrome*" | 5,061 |
| S20 | S3 AND S9 AND S18 AND S19 | 24 |

Bottom of Form

Bottom of Form

*Database*: Web of Science Core Collection

# 1

5,210,829

TOPIC: (infan* or newborn* or new-born* or perinat* or neonat* or baby* or babies or toddler* or minor* or boy* or girl* or kid or kids or child* or schoolchild* or adolescen* or juvenil* or youth* or teen* or pubescen* or pediatric* or paediatric* or peadiatric* or school* or prematur* or preterm* or "pre term*" or prepub* or pre-pub* or preschool* or pre-school* or kindergarten* or nursery* or preadolescen* or pre-adolescen*) OR TOPIC: ("young adult*" or "young person*" or "young people" or "young woman" or "young women" or "young man" or "young men")

# 2

2,937,703

TOPIC: ("Psychiatric Disorder*" or "Mental Disorder*" or Anxiety or Anxious or Phobia* or Phobic or Neurotic or Neurosis or "conversion disorder" or "medically unexplained symptoms" or PTSD or "Post Traumatic Stress Disorder" or Depress*) OR TOPIC: (Psychosis or Psychotic or "Cognition dysfunction" or "Cognitive function" or memory or "cognitive disorder" or "cognitive deficit" or "Executive function" or "Neuropsychological test*" or "Brain Disorder*" or "Mood Disorder*" or "Affective disorder*" OR Epilepsy or Epileptic or "Emotional Disorder") OR TOPIC: (Schizopreni* or Autism or ASD or Autistic or "Bipolar Disorder*" or "Mental* Ill*" or "Neurocognitive Disorder*" or "Neurodevelopmental Disorder*" or "Substance Abuse" or "Substance Misuse" or ADHD or "Attention deficit hyperactivity disorder" or "Behaviour* Disorder*" or "Behavior disorder") OR TOPIC: ("Emotional Disturbance*" or Suicid* or "Self Harm" or "Self Injur*" or "Self Destructive Behaviour" or "Self Destructive Behavior" or OCD or "Obsessive Compulsive Disorder" or "Neurological Disorder*" or "Neurological Disease*" or "Alcohol Misuse" or "Alcohol Abuse" or "Alcoholic" or "Conduct Disorder*" or "Oppositional Defiant Disorder" or paranoia or "hearing voices" or hallucination* or mania or manic or "eating disorder*" or "anorexia nervosa" or bulimi* or "binge eat*") OR TOPIC: ("mental health")

# 3

32,917

TOPIC: (phiv or (vertical* near/4 transmi*)) OR TOPIC: (perinatal* near/4 (infect* or Acquir* or transmi* or expos*)) OR TOPIC: (peri-natal* near/4 (infect* or Acquir* or transmi* or expos*)) OR TOPIC: (Fetomaternal* near/4 (infect* or Acquir* or transmi* or expos*)) OR TOPIC: ("mother to child" near/4 (infect* or Acquir* or transmi* or expos*)) OR TOPIC: ("maternal fetal" near/4 (infect* or Acquir* or transmi* or expos*)) OR TOPIC: (maternofetal near/4 (infect* or Acquir* or transmi* or expos*))

# 4

1,080,785

TOPIC: (hiv or "human immunodeficiency virus*" or hiv-1 or hiv-2 or "acquired immune deficiency syndrome*" or "human immune-deficiency virus*" or "acquired immunodeficiency syndrome*" or AIDS or "acquired immuno-deficiency syndrome*")

# 5

664

#4 AND #3 AND #2 AND #1

*Database*: Scopus

( ( TITLE-ABS-KEY ( infan* OR newborn* OR new-born* OR perinat* OR neonat* OR baby* OR babies OR toddler* OR minor* OR boy* OR girl* OR kid OR kids OR child* OR schoolchild* OR adolescen* OR juvenil* OR youth* OR teen* OR pubescen* OR pediatric* ) OR TITLE-ABS-KEY ( paediatric* OR peadiatric* OR school* OR prematur* OR preterm* OR "pre term*" OR prepub* OR pre-pub* OR preschool* OR pre-school* OR kindergarten* OR nursery* OR preadolescen* OR pre-adolescen* ) OR TITLE-ABS-KEY ( "young adult*" OR "young person*" OR "young people" OR "young woman" OR "young women" OR "young man" OR "young men" ) ) ) AND ( ( TITLE-ABS-KEY ( "Psychiatric Disorder*" OR "Mental Disorder*" OR anxiety OR anxious OR phobia* OR phobic OR neurotic OR neurosis OR "conversion disorder" OR "medically unexplained symptoms" OR ptsd OR "Post Traumatic Stress Disorder" OR depress* ) OR TITLE-ABS-KEY ( psychosis OR psychotic OR "Cognition dysfunction" OR "Cognitive function" OR memory OR "cognitive disorder" OR "cognitive deficit" OR "Executive function" OR "Neuropsychological test*" OR "Brain Disorder*" OR "Mood Disorder*" ) OR TITLE-ABS-KEY ( "Affective disorder*" OR epilepsy OR epileptic OR "Emotional Disorder" ) OR TITLE-ABS-KEY ( schizopreni* OR autism OR asd OR autistic OR "Bipolar Disorder*" OR "Mental* Ill*" OR "Neurocognitive Disorder*" OR "Neurodevelopmental Disorder*" OR "Substance Abuse" OR "Substance Misuse" OR adhd OR "Attention deficit hyperactivity disorder" ) OR TITLE-ABS-KEY ( "Behaviour* Disorder*" OR "Behavior disorder" ) OR TITLE-ABS-KEY ( "Emotional Disturbance*" OR suicid* OR "Self Harm" OR "Self Injur*" OR "Self Destructive Behaviour" OR "Self Destructive Behavior" OR ocd OR "Obsessive Compulsive Disorder" OR "Neurological Disorder*" OR "Neurological Disease*" ) OR TITLE-ABS-KEY ( "Alcohol Misuse" OR "Alcohol Abuse" OR "Alcoholic" OR "Conduct Disorder*" OR "Oppositional Defiant Disorder" OR paranoia OR "hearing voices" OR hallucination* OR mania OR manic OR "eating disorder*" OR "anorexia nervosa" OR bulimi* OR "binge eat*" ) OR TITLE-ABS-KEY ( "mental health" ) ) ) AND ( ( TITLE-ABS-KEY ( phiv OR ( vertical* W/4 transmi* ) ) OR TITLE-ABS-KEY ( perinatal* W/4 ( infect* OR acquir* OR transmi* OR expos* ) ) OR TITLE-ABS-KEY ( peri-natal* W/4 ( infect* OR acquir* OR transmi* OR expos* ) ) OR TITLE-ABS-KEY ( fetomaternal* W/4 ( infect* OR acquir* OR transmi* OR expos* ) ) OR TITLE-ABS-KEY ( "mother to child" W/4 ( infect* OR acquir* OR transmi* OR expos* ) ) OR TITLE-ABS-KEY ( "maternal fetal" W/4 ( infect* OR acquir* OR transmi* OR expos* ) ) OR TITLE-ABS-KEY ( maternofetal W/4 ( infect* OR acquir* OR transmi* OR expos* ) ) ) ) AND ( TITLE-ABS-KEY ( hiv OR "human immunodeficiency virus*" OR hiv-1 OR hiv-2 OR "acquired immune deficiency syndrome*" OR "human immune-deficiency virus*" OR "acquired immunodeficiency syndrome*" OR aids OR "acquired immuno-deficiency syndrome*" ) )

Database: **ProQuest Dissertations & Theses Global**

ab(infan* or newborn* or new-born* or perinat* or neonat* or baby* or babies or toddler* or minor* or boy* or girl* or kid or kids or child* or schoolchild* or adolescen* or juvenil* or youth* or teen* or pubescen* or pediatric* or paediatric* or peadiatric* or school* or prematur* or preterm* or "pre term*" or prepub* or pre-pub* or preschool* or pre-school* or kindergarten* or nursery* or preadolescen* or pre-adolescen* or "young adult*" or "young person*" or "young people" or "young woman" or "young women" or "young man" or "young men") AND ab("Psychiatric Disorder*" or "Mental Disorder*" or Anxiety or Anxious or Phobia* or Phobic or Neurotic or Neurosis or "conversion disorder" or "medically unexplained symptoms" or PTSD or "Post Traumatic Stress Disorder" or Depress* or Psychosis or Psychotic or "Cognition dysfunction" or "Cognitive function" or memory or "cognitive disorder" or "cognitive deficit" or "Executive function" or "Neuropsychological test*" or "Brain Disorder*" or "Mood Disorder*" or "Affective disorder*" OR Epilepsy or Epileptic or "Emotional Disorder" or Schizopreni* or Autism or ASD or Autistic or "Bipolar Disorder*" or "Mental* Ill*" or "Neurocognitive Disorder*" or "Neurodevelopmental Disorder*" or "Substance Abuse" or "Substance Misuse" or ADHD or "Attention deficit hyperactivity disorder" or "Behaviour* Disorder*" or "Behavior disorder" or "Emotional Disturbance*" or Suicid* or "Self Harm" or "Self Injur*" or "Self Destructive Behaviour" or "Self Destructive Behavior" or OCD or "Obsessive Compulsive Disorder" or "Neurological Disorder*" or "Neurological Disease*" or "Alcohol Misuse" or "Alcohol Abuse" or "Alcoholic" or "Conduct Disorder*" or "Oppositional Defiant Disorder" or paranoia or "hearing voices" or hallucination* or mania or manic or "eating disorder*" or "anorexia nervosa" or bulimi* or "binge eat*" or "mental health") AND ab(phiv or ("vertical* transmi*") or ((perinatal* or peri-natal* or Fetomaternal* or "mother to child" or "maternal fetal" or maternofetal) AND (infect* or Acquir* or transmi* or expos*))) AND ab(hiv or "human immunodeficiency virus*" or hiv-1 or hiv-2 or "acquired immune deficiency syndrome*" or "human immune-deficiency virus*" or "acquired immunodeficiency syndrome*" or AIDS or "acquired immuno-deficiency syndrome*")

Database: **World Health Organization Global Index Medicus** [**https://pesquisa.bvsalud.org/gim/**](https://pesquisa.bvsalud.org/gim/)

(tw:(((perinatal* or peri-natal* or Fetomaternal* or "mother to child" or "maternal fetal" or maternofetal) AND (infect* or Acquir* or transmi* or expos*)) OR phiv or ("vertical* transmi*"))) AND (tw:(hiv or "human immunodeficiency virus*" or hiv-1 or hiv-2 or "acquired immune deficiency syndrome*" or "human immune-deficiency virus*" or "acquired immunodeficiency syndrome*" or AIDS or "acquired immuno-deficiency syndrome*")) AND (tw:("Psychiatric Disorder*" or "Mental Disorder*" or Anxiety or Anxious or Phobia* or Phobic or Neurotic or Neurosis or "conversion disorder" or "medically unexplained symptoms" or PTSD or "Post Traumatic Stress Disorder" or Depress* or Psychosis or Psychotic or "Cognition dysfunction" or "Cognitive function" or memory or "cognitive disorder" or "cognitive deficit" or "Executive function" or "Neuropsychological test*" or "Brain Disorder*" or "Mood Disorder*" or "Affective disorder*" OR Epilepsy or Epileptic or "Emotional Disorder" or Schizopreni* or Autism or ASD or Autistic or "Bipolar Disorder*" or "Mental* Ill*" or "Neurocognitive Disorder*" or "Neurodevelopmental Disorder*" or "Substance Abuse" or "Substance Misuse" or ADHD or "Attention deficit hyperactivity disorder" or "Behaviour* Disorder*" or "Behavior disorder" or "Emotional Disturbance*" or Suicid* or "Self Harm" or "Self Injur*" or "Self Destructive Behaviour" or "Self Destructive Behavior" or OCD or "Obsessive Compulsive Disorder" or "Neurological Disorder*" or "Neurological Disease*" or "Alcohol Misuse" or "Alcohol Abuse" or "Alcoholic" or "Conduct Disorder*" or "Oppositional Defiant Disorder" or paranoia or "hearing voices" or hallucination* or mania or manic or "eating disorder*" or "anorexia nervosa" or bulimi* or "binge eat*" or "mental health")) AND (tw:(infan* or newborn* or new-born* or perinat* or neonat* or baby* or babies or toddler* or minor* or boy* or girl* or kid or kids or child* or schoolchild* or adolescen* or juvenil* or youth* or teen* or pubescen* or pediatric* or paediatric* or peadiatric* or school* or prematur* or preterm* or "pre term*" or prepub* or pre-pub* or preschool* or pre-school* or kindergarten* or nursery* or preadolescen* or pre-adolescen* or "young adult*" or "young person*" or "young people" or "young woman" or "young women" or "young man" or "young men"))

**OpenGrey** [**http://opengrey.eu**](http://opengrey.eu)

Child* AND mother AND transmi* AND hiv = 38

<http://opengrey.eu/search/request?q=child+AND+mother+AND+transmi*+AND+hiv>

child* AND vertical* AND transmi* AND hiv = 8

<http://opengrey.eu/search/request?q=child*+AND+vertical*+AND+transmi*+AND+hiv>

child* AND maternal* AND transmi* AND hiv = 4

<http://opengrey.eu/search/request?q=child*+AND+maternal*+AND+transmi*+AND+hiv>

child* AND maternofetal* AND hiv = 0

<http://opengrey.eu/search/request?q=child*+AND+maternofetal*+AND+hiv>

child* AND fetomaternal* AND hiv = 0

<http://opengrey.eu/search/request?q=child*+AND+fetomaternal*+AND+hiv>

child* AND perinatal* AND hiv = 2

<http://opengrey.eu/search/request?q=child*+AND+perinatal*+AND+hiv>

## S2- PRISMA flow diagram

**Results**

## S3- Newcastle Ottowa Risk of Bias Table

**SELECTION** (one star only for each question)

1. Is the case definition adequate/ how did they ascertain HIV status (in all groups)?
   1. Reference to primary medical record ☼
   2. Lab confirmation of HIV status ☼
   3. Self-report
   4. No description
2. Representativeness of the PHIV group
   1. Truly represent the average child with pHIV in the study area – e.g all or a random sample of children attending a HIV clinic or antenatally recruited, without significant loss to follow up or non-response rate from cohort which may have introduced selection bias ☼
   2. Somewhat representative - not random but SES, ethnicity etc. is representative, without significant loss to follow up or non-response rate which may have introduced selection bias ☼
   3. Potential for selection bias e.g. convenience sample, self-selected, sick children in hospital, high non-response rate if part of a cohort, significant loss to follow up
   4. Not stated
3. Selection of non-exposed groups (HIVEU & HIVUU group)
   1. Same community as cohort – e.g. same family, same clinic, same social group, without significant loss to follow up or non-response rate which may have introduced selection bias ☼
   2. Hospital controls or from a different source, non-response rate or loss to follow up introducing selection bias, not similar to pHIV group.
   3. No description
4. Sample size
   1. Sample size justified & adequately powered
   2. No justification or not adequately powered

**COMPARABILITY (2 stars)**

1. Confounding factors were taken into account in case-matching or adjusted for in analysis (can get 2 stars for this Q)
   1. Socioeconomic status ☼
   2. Analysis/ matching accounted for at least one of the following ☼:
      1. Orphanhood or primary caregiver not a parent
      2. Perinatal factors/ other drugs
      3. Age or sex
      4. Ethnicity
      5. Schooling
   3. None of the above

**OUTCOME** (1 star only for each question)

1. Ascertainment of outcome
   1. Validated neuropsych scale for the population where the assessor was trained, and blinded to HIV status, or a was self/caregiver-reported scale, or neuropsych outcomes ascertained from secure medical record, **the same in all groups**☼
   2. Un-blinded assessment or un-validated scale or different in different groups
   3. No description
2. Statistical test
   1. The statistical test used to analyse the data is clearly described and appropriate, and the measurement of the association is presented with size of effect, confidence intervals/ standard deviation and p values☼
   2. Not described or not appropriate

Very good 7-8, Good 5-6, Adequate 3-4, Poor

| **AUTHOR** | **YEAR** | **STUDY TYPE** | **SELECTION** | | | | | | | | **COMPARABILITY** | | **OUTCOME** | | | | **TOTAL** | **SCORE** |
| --- | --- | --- | --- | --- | --- | --- | --- | --- | --- | --- | --- | --- | --- | --- | --- | --- | --- | --- |
|  |  |  | **1** | ***** | **2** | ***** | **3** | ***** | **4** | ***** | 1 | ** | 1 | * | 2 | * |  |  |
| Chase | 1995 | cross-sectional | a | 1 | a | 1 | a | 1 | b | 0 | ab | 2 | b | 0 | a | 1 | 6 | good |
| Gay | 1995 | cross-sectional | a | 1 | a | 1 | a | 1 | b | 0 | ab | 2 | b | 0 | a | 1 | 6 | good |
| Bovin | 1995 | cross-sectional | b | 1 | c | 0 | b | 0 | b | 0 | ab | 2 | b | 0 | a | 1 | 4 | adequate |
| Drotar | 1997 | cross-sectional | a | 1 | a | 1 | a | 1 | b | 0 | ab | 2 | b | 0 | a | 1 | 6 | good |
| Chase | 2000 | cohort | b | 1 | b | 1 | a | 1 | b | 0 | b | 1 | a | 1 | a | 1 | 6 | good |
| Bruck | 2001 | cross-sectional | b | 1 | c | 0 | c | 0 | b | 0 | c | 0 | a | 1 | a | 1 | 3 | adequate |
| Mcgrath | 2006 | cohort | b | 1 | a | 1 | a | 1 | b | 0 | c | 0 | a | 1 | a | 1 | 5 | good |
| Whitehead | 2014 | cohort | b | 1 | c | 0 | a | 1 | a | 1 | c | 0 | b | 0 | a | 1 | 4 | adequate |
| McHenry | 2021 | cross sectional pilot | c | 0 | c | 0 | b | 0 | b | 0 | c | 0 | a | 1 | a | 1 | 2 | poor |
| Puthanakit | 2010 |  | a | 1 | b | 1 | b | 0 | b | 0 | ab | 2 | b | 0 | a | 1 | 5 | good |
| Smith | 2012 | PHACS AMP | c | 0 | b | 1 | a | 1 | b | 0 | ab | 2 | b | 0 | a | 1 | 5 | good |
| Boivin | 2019 | IMPACT p1060 | c | 0 | b | 1 | b | 1 | a | 1 | ab | 2 | b | 0 | a | 1 | 6 | good |
| Debeaudrap | 2018 | Pediacam ANRS12140 cohort | b | 1 | c | 0 | b | 0 | b | 0 | ab | 2 | b | 0 | a | 1 | 4 | adequate |
| Cohen | 2015 | NOVICE | b | 1 | a | 1 | c | 0 | b | 0 | ab | 2 | b | 0 | a | 1 | 5 | good |
| Ghate | 2015 |  | d | 0 | c | 0 | b | 0 | b | 0 | b | 1 | b | 0 | b | 0 | 1 | poor |
| Judd | 2016 | AALPHI | a | 1 | a | 1 | a | 1 | b | 0 | ab | 2 | b | 0 | a | 1 | 6 | good |
| Blanchette | 2002 |  | b | 1 | c | 0 | a | 1 | b | 0 | a | 1 | b | 0 | a | 1 | 4 | adequate |
| Chernoff | 2009 | IMPACT 1055 | a | 1 | b | 1 | a | 1 | b | 0 | ab | 2 | b | 0 | a | 1 | 6 | good |
| Lee | 2011 |  | a | 1 | c | 0 | b | 0 | b | 0 | b | 1 | b | 0 | a | 1 | 3 | adequate |
| Mellins | 2012 | CASAH | a | 1 | c | 0 | b | 0 | b | 0 | ab | 2 | b | 0 | a | 1 | 4 | adequate |
| Louthrenoo | 2014 |  | a | 1 | b | 1 | c | 0 | b | 0 | ab | 2 | b | 0 | a | 1 | 5 | good |
| Louw | 2016 |  | a | 1 | c | 0 | b | 0 | b | 0 | b | 1 | a | 1 | a | 1 | 4 | adequate |
| Mutumba | 2016 | CASAH | a | 1 | c | 0 | a | 1 | b | 0 | ab | 2 | b | 0 | a | 1 | 5 | good |
| Abubakar | 2017 |  | c | 0 | c | 0 | b | 0 | b | 0 | ab | 2 | b | 0 | a | 1 | 3 | adequate |
| Le Prevost | 2018 | AALPHI | a | 1 | a | 1 | a | 1 | b | 0 | ab | 2 | a | 1 | a | 1 | 7 | good |
| Rukuni | 2018 |  | b | 1 | c | 0 | b | 0 | b | 0 | b | 1 | b | 0 | a | 1 | 3 | adequate |

## S4- Young People’s Advisory Group (YPAG) HIV

The following questions were put to the group:

A lot of studies on HIV, but fewer on children and on the long-term effects

We have looked at all the studies on children who have had HIV their whole life

There were 92 papers which had a lot of detail

We were interested in their learning and school and their anxieties and worries

We want to know what young people like you would like to know

Are there other people who we should tell?

**What do you think young people who have grown up with HIV might like to know?**

- Mary is 13 and has been coming to this clinic for many years
- She is quiet and shy
- She never asks many questions
- The last time she came she seemed worried about her foot and reluctant to leave, but it was unclear what was going on.
- How could the people in clinic best help Mary say what she is worried about?
- What do you think Mary might know about her HIV? Where might she have heard this information?
- What might be worrying her today?
- Who else might she be able to ask?
- What would help her to ask the questions that are worrying her? (which might be nothing to do with her foot)

What might she be worrying about with her HIV in general

Have you ever had a question about your HIV that you have found difficult to ask? Yes/no/can’t remember

- If we find that, for example, children who grow up with HIV find reading more difficult or can struggle with worries, would young people want to know this?
- How would you tell the young people?
- Who else would you like to know?
  1. How should we tell them?

## The following information was collected at the YPAG:

Location: Young people with Dr Nyasha from Chiedza trial in Harare, Zimbabwe

Rebecca Horton, Mina Fazel remotely

12 children and young people with perinatally acquired HIV who are part of the Chiedza trial. Aged between 11 and 18 years. Three guardians were present and also contributed to the meeting.

Meeting lasted 2 hours total.

Translation between Shona and English provided by Dr Nyasha

Themes which emerged:

- Stigma around HIV diagnosis still very prominent and has significant impact on the lives of young people (not wanting to disclose to teachers, reports of abuse from teachers). Came up repeatedly throughout the group. A participant expressed a wish for legal penalties for teachers who discriminated against children for HIV status
- Poor perceived knowledge of peers about HIV – participants wished that peers knew that HIV was not only from prostitution, that a diagnosis of HIV did not define the individual
- Young people were very engaged in clinic, clinic and peer counsellors major source of advice and support
- Generally a lot of comments about the importance of taking ARVs, but awareness of reasons why peers may default treatment

Worries about neurocognitive impact of HIV

- Generally, fewer concerns about physical impact of HIV on the body than of stigma/ psychosocial effects
- Stroke and learning disability were mentioned
- Mental illness mentioned
- Young person commented that ‘ it is the environment which is toxic to the brain, not the virus’ – stating that the environment/stigma is more likely to cause the learning disability rather than the direct effects of HIV- stigma/lower expectations of them
- Concerns raised about ‘memory problems’
- Concerns about mood fluctuations
- One guardian stated that she felt her daughter had some neurocognitive difficulties and was slow to process commands.

Other worries

- Skin concerns were raised
- Mentioned that stopping ARVs does not lead to immediate symptoms, so that if someone regularly misses them because they don’t want to take them when with friends, this could lead to regularly missing treatment and disease progression

Dissemination

- Participants mostly got information through clinic
- Stated that whilst they would rather hear it from doctors/nurses/ counsellors at the clinic and that new research info should be directed to them, young people who were less engaged might benefit from other sources
- Posters (in Harare centre, at youth centres, at churches) were popular
- Radio stations (no consensus reached on which)
- Social media was not a popular information source
- Generally, participants had not disclosed their status to their teachers and therefore additional HIV research information not helpful for teachers
- One guardian mentioned that she had disclosed her daughter’s status to her teacher, in order that she could not get in trouble for being slow to follow commands (which guardian attributed to her HIV). Participant said it meant the teacher was understanding of her slower progress, but that there had been some incidents where the teacher was talking about HIV and she was worried that she was talking about her.
- Guardians expressed wanting to know about any possible complications as early as possible so that they can seek help
- ‘Road shows’ were mentioned

How can doctors/nurses help address worries?

- Expressed preference for peer counsellors- other people with HIV, young in age
- Mixed opinions over whether the doctor/nurse was the same gender as them
- ‘Ok if they don’t have HIV if they listen well and are kind’
